# Mutations in unfolded protein response regulator ATF6 cause hearing and vision loss syndrome

**DOI:** 10.1172/JCI175562

**Published:** 2025-02-03

**Authors:** Eun-Jin Lee, Kyle Kim, Monica Sophia Diaz-Aguilar, Hyejung Min, Eduardo Chavez, Korina J. Steinbergs, Lance A. Safarta, Guirong Zhang, Allen F. Ryan, Jonathan H. Lin

**Affiliations:** 1 Departments of Pathology and; 2 Ophthalmology, Stanford University School of Medicine, Stanford, California, USA.; 3 VA Palo Alto Healthcare System, Palo Alto, California, USA.; 4 Rush University Medical College, Chicago, Illinois, USA.; 5 Departments of Otolaryngology and Neuroscience, UCSD and Veterans Administration Medical Center, La Jolla, California, USA.

**Keywords:** Genetics, Ophthalmology, Genetic diseases, Molecular genetics, Retinopathy

## Abstract

Activating transcription factor 6 (ATF6) is a key regulator of the unfolded protein response (UPR) and is important for ER function and protein homeostasis in metazoan cells. Patients carrying loss-of-function *ATF6* disease alleles develop the cone dysfunction disorder achromatopsia. The effect of loss of ATF6 function on other cell types, organs, and diseases in people remains unclear. Here, we report that progressive sensorineural hearing loss was a notable complaint in some patients carrying *ATF6* disease alleles and that *Atf6^–/–^* mice also showed progressive auditory deficits affecting both sexes. In mice with hearing deficits, we found disorganized stereocilia on hair cells and focal loss of outer hair cells. Transcriptomics analysis of *Atf6^–/–^* cochleae revealed a marked induction of the UPR, especially through the protein kinase RNA-like endoplasmic reticulum kinase (PERK) arm. These findings identify ATF6 as an essential regulator of cochlear health and function. Furthermore, they support the idea that ATF6 inactivation in people causes progressive sensorineural hearing loss as part of a blindness-deafness genetic syndrome targeting hair cells and cone photoreceptors. Last, our genetic findings indicate that ER stress is an important pathomechanism underlying cochlear damage and hearing loss, with clinical implications for patient lifestyle modifications that minimize environmental and physiological sources of ER stress to the ear.

## Abstract 2

ATF6 inactivating patient mutations cause a blindness-deafness genetic syndrome characterized by cone photoreceptor dysfunction achromatopsia and sensorineural hearing loss.

## Introduction

Activating transcription factor 6 (ATF6) encodes a glycosylated 670–amino acid type II ER transmembrane protein that regulates a signal transduction pathway of the unfolded protein response (UPR) (1, 2). In response to ER stress, ATF6 protein migrates from the ER to the Golgi apparatus where Golgi-resident site 1 and site 2 proteases cleave the ATF6 transmembrane domain and liberate the cytosolic bZIP transcription factor domain (3, 4). The liberated ATF6 bZIP-transcription factor domain enters the nucleus and upregulates ER chaperones, ER protein folding enzymes, and ER-associated degradation (ERAD) components (1, 5–8). Thus, ATF6 signaling helps cells adapt to ER stress by enhancing ER protein folding functions and maintaining ER protein quality (2, 9).

In people, we previously identified many ATF6 alleles that disrupt ATF6 signaling (10–13). These include missense variants in the luminal domain of ATF6 that impair ER-to-Golgi trafficking of the full-length ATF6 protein (11, 12); missense variants in the bZIP DNA-binding domain of ATF6 (10, 11); premature stop codons and splice-site variants (10, 13); as well as large multi-exon deletions at the ATF6 locus (13). Fibroblasts and stem cells from people carrying these ATF6 alleles and expression of recombinant ATF6 variant proteins in HEK293 cells all revealed loss of ATF6 transcriptional activity (10, 13–15). Individuals who are homozygous or compound heterozygous for these variants have congenital vision loss diseases, achromatopsia, and cone-rod dystrophy (10, 12, 13, 16, 17). Patient retinas and retinal organoids differentiated from patient induced pluripotent stem cells (iPSCs) or from *Atf6^–/–^* human embryonic stem cells (hESCs) generated by CRISPR deletion revealed an absence of outer segments, the organelle responsible for phototransduction, on cone photoreceptors, and this subcellular defect underpins the photopic vision loss in these patients (15, 18). To our knowledge, no other diseases or cellular defects have been reported in people lacking ATF6 function.

The absence of ATF6 has a notable effect across a number of organs in various organisms and experimental disease models. In the eyes, *Atf6^–/–^* mice exhibit late-onset retinal degeneration and accelerated degeneration when bred with mice of the P23H rhodopsin model of retinitis pigmentosa (10, 19). In the liver and pancreas, *Atf6^–/–^* mice and zebrafish with ATF6 morpholinos demonstrate symptoms like steatosis, fatty liver, β cell loss, obesity, and type 2 diabetes–like features after intraperitoneal tunicamycin injection (7, 20), and high-fat diet feeding (21) when bred with Agouti and Akita diabetes mouse models (21), or when treated with ethanol (22). In the cardiovascular system, *Atf6^–/–^* mice show myocardial damage and reduced cardiac function in models of ischemic heart disease (23, 24) and increased brain infarction in stroke models (25). In muscle, *Atf6^–/–^* mice show increased myofiber damage after treadmill exercise challenge (26). In the colon, *Atf6^–/–^* mice develop severe colitis following dextran sulfate sodium administration (27). In neurological contexts, *Atf6^–/–^* mice demonstrate increased neuronal cell death in models of Parkinson’s disease (28) and glutamate excitotoxicity (29), as well as hypothalamic neuron defects affecting water balance and urine production (30). Interestingly, the loss of ATF6 has some beneficial effects: *Atf6^–/–^* mice resist paralysis and show reduced spinal cord inflammation in a model of multiple sclerosis (31). *Atf6^–/–^* mice also display no notable differences from controls in an osteoarthritis model (32). Notably, under normal laboratory growing conditions, *Atf6^–/–^* mice were viable and showed none of the above changes except for the retinal decline seen with advanced age (7, 10).

In light of the broad range of organs affected by a loss of ATF6 in animal models, in this study, we investigated whether patients lacking functional ATF6 had additional diseases or phenotypes other than the previously reported vision loss. Unexpectedly, we found that progressive sensorineural hearing loss was a prominent complaint in our patient cohorts. Furthermore, we identified a previously unappreciated auditory defect in *Atf6^–/–^* mice that phenocopied the hearing loss found in patients. Then, we characterized the cellular and molecular defects in the ear underlying hearing loss in the *Atf6^–/–^* mice.

## Results

### Human ATF6 mutations are associated with hearing loss.

**Figure 1:**
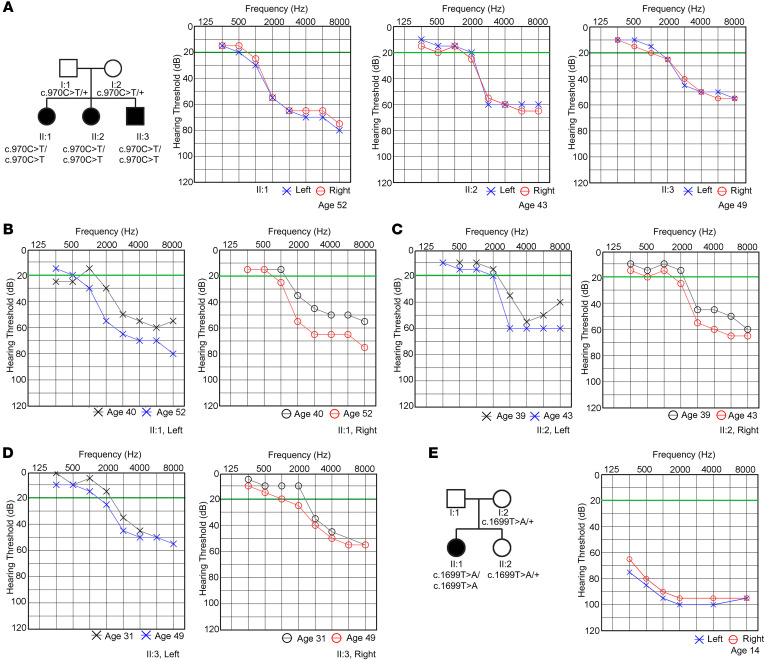
Human *ATF6* mutations are linked to hearing loss. (**A**) Pedigree of the family carrying the point mutation (c.970C>T, p.Arg324Cys) and audiograms from individuals aged 52, 43, and 49 years showing moderate high-frequency hearing loss in both the right (red line) and left (blue line) ears. (**B**) Audiograms from patient II:1 at ages 40 and 52 years revealed progressive high-frequency hearing loss. (**C**) Audiograms from patient II:2 at ages 39 and 43 years show a similar hearing loss progression. (**D**) Audiograms from patient II:3 at ages 31 and 49 years show some progression of high-frequency hearing loss. (**E**) Pedigree of the family carrying the point mutation (c.1699T>A, p.Tyr567Asn, age 14 years) shows severe low- to high-frequency hearing loss in both the right (red line) and left (blue line) ears. Green line indicates a normal hearing threshold.

Hearing loss was a prominent complaint in multiple patients with achromatopsia carrying ATF6 disease variants. Hearing loss was reported by 3 achromatopsia siblings homozygous for a c.970C>T ATF6 variant that introduced an arginine-to-cysteine conversion at position 324 (Figure 1A, II:1, II:2, and II:3). This missense mutation abrogated ATF6 transcriptional activity in cultured fibroblasts from these siblings when challenged with tunicamycin and thapsigargin (10, 11). In all 3 individuals, audiometry testing showed hearing defects most evident in high-frequency ranges that affected the right and left ears equally (Figure 1A, audiograms from patient II:1 at age 52, patient II:2 at age 43, and patient II:3 at age 49). Otoacoustic emissions were absent in patients II:1 and II:2. Patient II:3 showed some response but only up to 3 kHz on the right and 2 kHz on the left and an unusually high response amplitude in the 5 kHz band on the left (10 decibels sound pressure level [dB SPL]) (Supplemental Figure 1; supplemental material available online with this article; https://doi.org/10.1172/JCI175562DS1). The results indicated a loss of outer hair cells (OHCs) in the area from 3–5 kHz in the left ear. However, there was evidence of a narrow island of surviving hair cells around the 5 kHz region in the left ear, which the audiologist marked as an anomalous finding. Hearing was not tested at this frequency, so some function may have been missed.

Comparison of audiograms performed at different time points revealed a progressive worsening of the hearing loss affecting both ears (Figure 1B, left and right audiograms from patient II:1 performed at age 40 and age 52, and Supplemental Figure 2A). Figure 1C shows bilateral audiograms from patient II:2 performed at age 39 and age 43 (Supplemental Figure 2B). Figure 1D shows bilateral audiograms from patient II:3 performed at age 31 and age 49. Medical genetic testing of blood samples from these 3 patients showed no pathogenic variants in a 356-gene hereditary hearing loss panel (Supplemental Table 1). Commercial gene testing of saliva from patient II:3 also revealed no known genetic basis for hearing loss (23andMe ancestry test). Hearing loss was also reported in another patient (II:1) with achromatopsia who was homozygous for a c.1699T>A ATF6 variant that introduced a tyrosine-to-asparagine conversion at position 567 (Figure 1E). This missense mutation also abrogated ATF6 transcriptional activity in fibroblasts challenged with tunicamycin and thapsigargin in vitro (10, 11). Audiometry testing showed auditory defects affecting the right and left ears equally (Figure 1E, audiogram at age 14). Genetic testing for hereditary hearing loss in patient II:1 (Figure 1E) was not conducted, as consent for genetic testing was not obtained. Additionally, progressive audiometric assessments were not conducted for this patient. Nonetheless, audiometric findings in individuals with ATF6 variants linked to disease, coupled with the absence of mutations in known hereditary sensorineural genes in at least 3 of these cases, suggested that ATF6 may be causative for sensorineural hearing loss.

### Atf6^–/–^ mice exhibit hearing loss.

**Figure 2:**
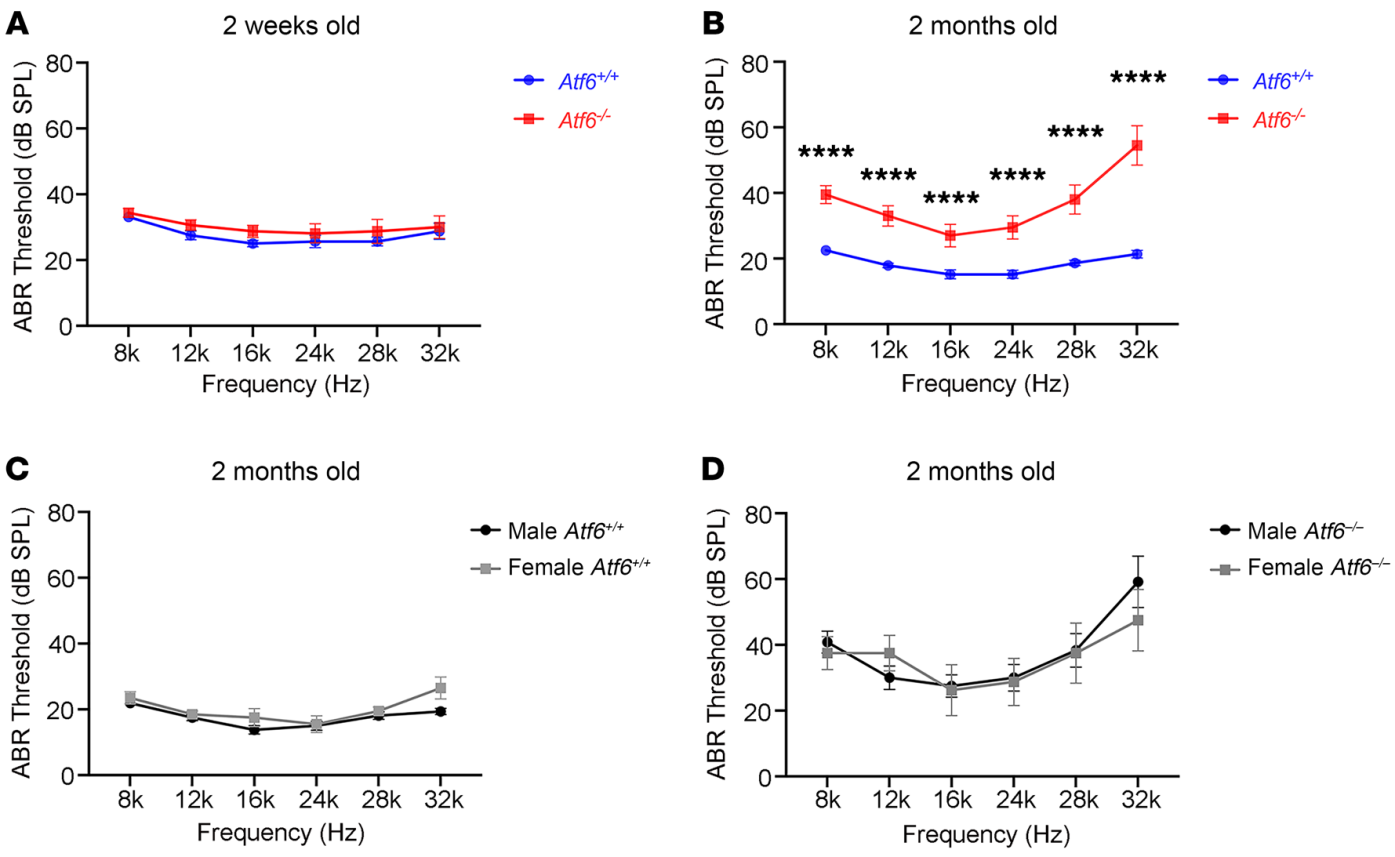
Hearing loss in *Atf6^–/–^* mice. (**A**) At P14 (2 weeks old), ABR thresholds at 6 frequencies were indistinguishable between *Atf6^+/+^* (*n* = 8, blue line) and *Atf6^–/–^* (*n* = 8, red line) mice. Data represent the mean ± SEM. *P* > 0.05, 2-way ANOVA. (**B**) At 2 months of age, *Atf6^–/–^* mice (*n* = 10, red line) showed statistically significantly increased ABR thresholds (consistent with profound hearing loss) at all frequencies compared with *Atf6^+/+^* mice (*n* =13, blue line). Data represent the mean ± SEM. ****P ≤ 0.001, 2-way ANOVA. (**C** and **D**) No sex differences in ABR thresholds between 2-month-old female (gray line) and male (black line) *Atf6^+/+^* (*n* = 8, male; *n* = 5 female) and *Atf6^–/–^* (*n* = 6, male; *n* = 4 female) mice. Data represent the mean ± SEM. *P* > 0.05, 2-way ANOVA.

To further investigate ATF6’s role in hearing, we analyzed auditory responses in *Atf6*^–/–^ mice. These mice carry a premature stop codon in exon 4 and produce no ATF6 protein, similar to some ATF6 disease alleles found in patients with achromatopsia (ACHM) (7, 13). *Atf6*^–/–^ mice are viable and have normal growth and development but display functional changes in many experimental ER stress–linked disease paradigms (7, 23–28, 30). To our knowledge, their hearing has not been studied. We evaluated auditory brainstem responses (ABRs) to assess auditory function in the absence of ATF6. First, we measured auditory function at P14, (i.e., 2 weeks), shortly after the onset of hearing function (33). In 2-week-old mice raised under normal vivarium conditions, measurements of ABRs to pure tones showed no statistically significant changes in ABR thresholds (measured in dB SPL) at all tested frequencies (i.e., 8, 12, 16, 24k, 28, and 32 kHz) in *Atf6*^+/+^ versus *Atf6*^–/–^ mice (Figure 2A). However, by 2 months, the ABR thresholds of *Atf6*^–/–^ mice were statistically significantly higher than those of *Atf6*^+/+^ mice at all tested frequencies (****P ≤ 0.0001, 2-way ANOVA), with the highest thresholds at 32 kHz (high-frequency stimuli, Figure 2B). We detected no significant sex differences in ABR thresholds in the *Atf6*^–/–^ or *Atf6*^+/+^ mice (*P* > 0.05, 2-way ANOVA) (Figure 2, C and D) in the ABR tests done in these mice at 2 months of age. These findings demonstrate that *Atf6*^–/–^ mice had normal hearing at the onset of hearing (i.e., 2 weeks of age) but developed an auditory defect by 2 months of age.

Our transgenic *Atf6^–/–^* (7, 10, 19) mice were maintained on the C57BL/6J (B6J) genetic background. The B6J mouse strain carries the defective *Cdh23ahl* allele and develops age-related hearing loss beginning at approximately 3–6 months with worsening over the next 12–15 months (33–37). We assessed auditory structure and function in *Atf6^–/–^* mice at 2 months of age because experiments in older mice would be confounded by the inherent hearing loss that occurs in mice on the B6J genetic background. Thus, our results suggest that the hearing loss in *Atf6^–/–^* mice was not due to the B6J background because all the recordings were performed in 2-week-old and 2-month-old mice. Next, we investigated cellular and molecular defects responsible for hearing loss in the *Atf6*^–/–^ mice.

### Normal inner and outer ear anatomy and no inflammation in Atf6^–/–^ mouse ears.

**Figure 3:**
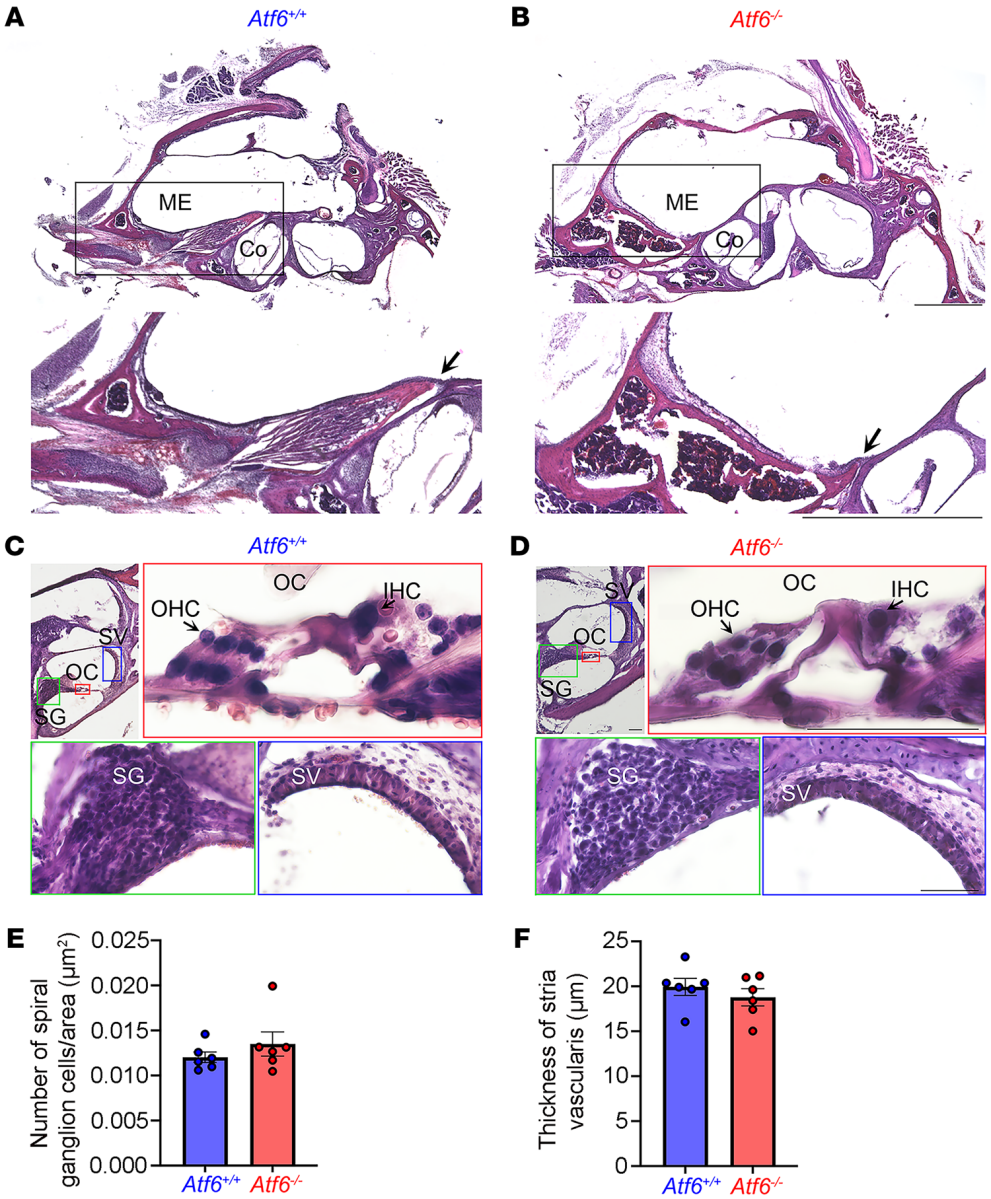
Histology of *Atf6^+/+^* and *Atf6^–/–^* MEs and IEs. (**A** and **B**) Light micrographs taken from cryostat sections and processed for H&E staining of ME tissue of 2-month-old *Atf6^+/+^* and *Atf6^–/–^* mice show normal anatomy and no infection in the ME. The bottom image represents higher magnification of the boxed region of the ME and shows no accumulation of inflammatory exudate extending from the eustachian tube (arrows) or in the ME cavity. (**C** and **D**) Histologic sections of the OC (red box) derived from 2-month-old *Atf6^+/+^* and *Atf6^–/–^* mice show nondisrupted anatomic architecture of the *Atf6^–/–^* cochlea as well as an intact SV (blue box), SG cells (green box), OHCs, and IHCs. (**E**) The number of SP cells/area and (**F**) the thickness of the SV were not statistically significantly different between *Atf6^+/+^* (*n* = 6) and *Atf6^–/–^* (*n* = 6) cochleae. Dots represent individual measurements. Data represent the mean ± SEM. *P* > 0.05, Welch’s *t* test. Scale bars: 1 mm (**A** and **B**); 1 mm (**A** and **B**, insets); 50 μm (**C** and **D**, including insets).

Bacterial infections of the middle ear (ME), or otitis media, affect 80% of the human population and can lead to chronic infection, inflammation, scarring, and tissue damage in the ME, leading to hearing loss (38–45). To determine whether *Atf6*^–/–^ mice had infection, inflammation, scarring, or tissue damage in the ME, we analyzed prepared histologic sections of the ears from 2-month-old *Atf6*^–/–^ mice. Neither *Atf6*^+/+^ nor *Atf6*^–/–^ mice showed infectious or inflammatory exudate extending from the eustachian tube (arrows, Figure 3, A and B), and the ME was open, patent, and free of inflammatory cells in all mice (Figure 3, A and B). Furthermore, no scarring or tissue damage was noted in the MEs of *Atf6*^+/+^ and *Atf6*^–/–^ mice. These findings support that hearing loss in *Atf6*^–/–^ does not arise from ME inflammation and structural destruction.

Next, we examined the anatomy of the cochlea within the inner ear (IE) in *Atf6*^–/–^ mice. The cochlea converts sound into sensorineural impulses transmitted to the brain to provide auditory information. Damage to the cochlear epithelial or sensorineural tissues such as the stria vascularis (SV), the organ of Corti (OC), or spiral ganglion (SG) cells can cause hearing loss (46–50). On prepared histologic sections of the cochlea from 2-month-old *Atf6*^+/+^ (Figure 3C) and *Atf6*^–/–^ mice (Figure 3D), we found normal cochlear anatomy in *Atf6*^–/–^ mice with intact SV and SG cells, as in *Atf6*^+/+^ mice. Consistent with the histologic appearance, quantification of the number of SG cells/area (Figure 3E, *P* = 0.99, Welch’s *t* test) and the thickness of the SV (Figure 3F, *P* = 0.86, Welch’s *t* test) revealed no statistically significant differences between *Atf6*^+/+^ and *Atf6*^–/–^ mice. Therefore, these results indicate that the hearing defect in *Atf6*^–/–^ mice was unlikely to have arisen from damage to SG cells or atrophy of the SV in the cochlea. In these histologic preparations, the OC also appeared structurally normal in *Atf6*^–/–^ mice, with discernible IHCs and OHCs separated by a tunnel (Figure 3, C and D), but stereocilia and other subcellular morphology of hair cells could not be resolved with this method.

### Atf6^–/–^ cochleae show loss of hair cells and disorganized hair cell stereocilia.

**Figure 4:**
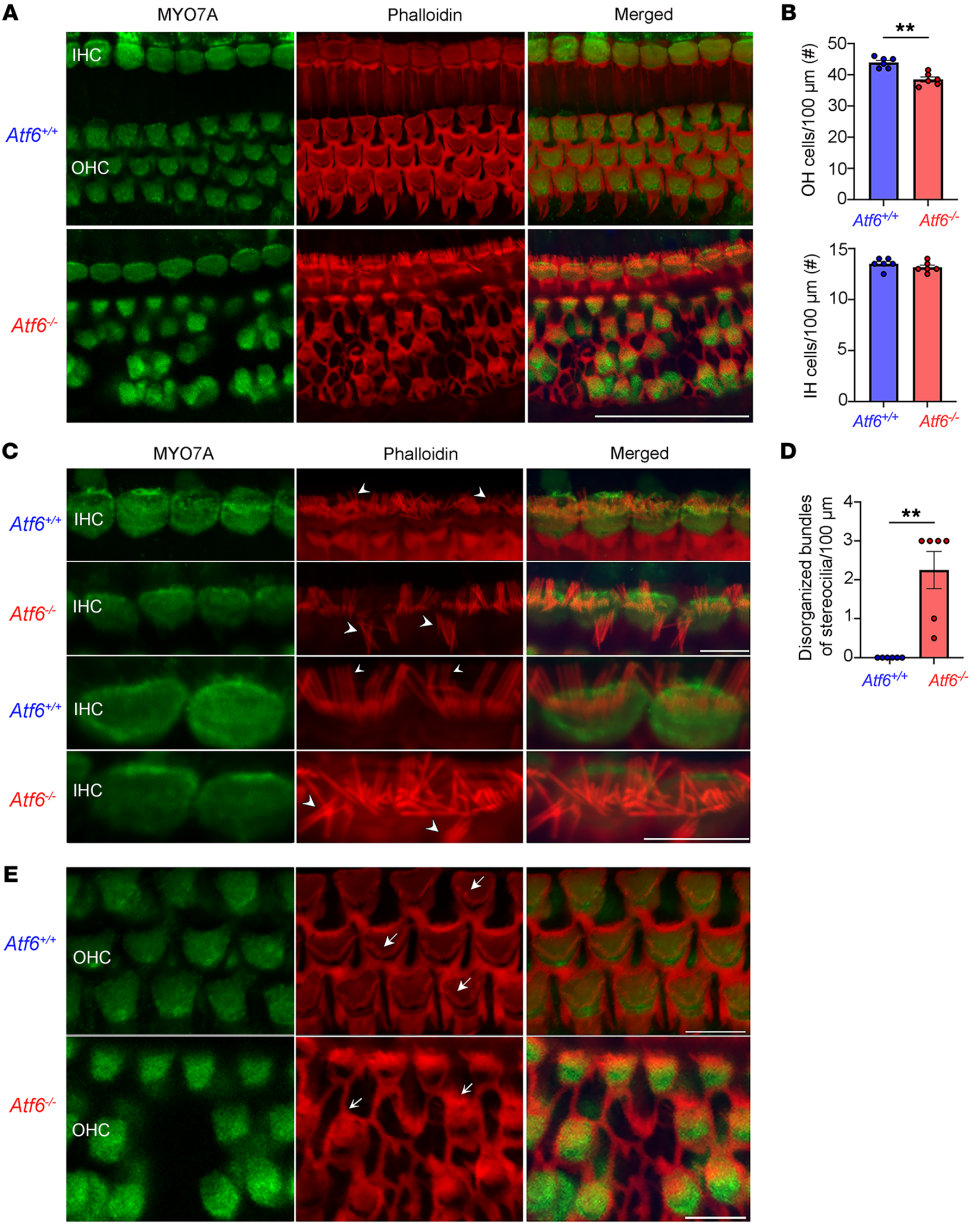
*Atf6^–/–^* mouse cochlear hair cells degenerate and exhibit disorganized stereocilia in the basal region at an approximate frequency of 28–32 kHz. *Atf6^+/+^* and *Atf6^–/–^* cochleae were stained for myosin 7a (MYO7A) and phalloidin to visualize hair cells and stereocilia, respectively. (**A**) Immunofluorescence confocal images of 2-month-old *Atf6^–/–^* OHCs show disorganized arrangement and loss of OHCs. (**B**) Histogram showing the number of OHCs and IHCs in 2-month-old *Atf6^+/+^* (*n* = 6) and *Atf6^–/–^* (*n* = 6) cochleae. Counts refer to the number of hair cells encountered within the average of 3 separate 100 linear extensions. Each dot represents the average of 2 individual measurements. Data represent the mean ± SEM. **P ≤ 0.01, Welch’s *t* test. (**C**) *Atf6^+/+^* mice maintained organized stereocilia on IHCs, whereas *Atf6^–/–^* mice had IHC stereocilia abnormalities such as disorganized bundling (arrowheads) at 2 months of age. Bottom 2 rows are higher-power images showing stereocilia organization in *Atf6^+/+^* versus *Atf6^–/–^* IHCs. (**D**) Quantitative analysis of disorganized IHC bundle reveals statistically significant disorganization of stereocilia in 2-month-old *Atf6^–/–^* mice. Dots represent individual measurements (*n* = 6). Data represent the mean ± SEM. **P ≤ 0.01, Welch’s *t* test. (**E**) Images of MYO7A and phalloidin staining of OC tissue from *Atf6^+/+^* and *Atf6^–/–^* cochleae focusing on the stereocilia of the OHCs. In *Atf6^–/–^* mice (*n* = 6), OHC stereocilia show severe changes in morphology when compared with OHC stereocilia from *Atf6^+/+^* mice (arrows, *n* = 6). Scale bars: 50 μm (**A**) and 10 μm (**C** and **E**).

Hair cells use stereocilia bundles at their apex to detect sound wave pressure changes in the endolymph (48). Damage to hair cell structures decreases hearing sensitivity because hair cells cannot regenerate in mice or humans (51–54). To evaluate hair cells in detail, we prepared whole mounts of cochleae from *Atf6*^–/–^ mice (55). Immunofluorescence with a hair cell–specific marker, myosin VII (56–58), and a filamentous actin marker, phalloidin (58, 59), were used to evaluate the cellular arrangement and the stereocilia of hair cells in *Atf6*^+/+^ and *Atf6*^–/–^ mice (Figure 4). At 2 months, there was apparent cell loss and disrupted arrangement of OHCs in the basal region of the cochlea (Figure 4, A and B, *P* = 0.02, Welch’s *t* test, Supplemental Figure 3), but no cell loss was found among the inner hair cells (IHCs) in the cochlea (Figure 4, A and B, *P* = 0.28, Welch’s *t* test*)*. In contrast to the OHC loss in the cochlear base, the OHCs in the apical region appeared to be preserved (Supplemental Figure 4).

The proper orientation, projection, and alignment of mechanosensory stereocilia on the apical surfaces of hair cells are essential for hearing. Actin labeling of the stereocilia bundles revealed severe disorganization of these structures in *Atf6*^–/–^ mice (Figure 4). In the basal cochlea, many stereocilia on IHCs were misaligned and extended in random directions (Figure 4, C and D, *P* = 0.002, Welch’s *t* test) in *Atf6*^–/–^ mice (arrowheads, Figure 4C) compared with the *Atf6*^+/+^ mice. In this region of the cochlea, surviving OHCs also showed disorganized stereocilia (arrows, Figure 4E) in *Atf6*^–/–^ mice. In contrast to the stereocilia damage seen in IHCs and OHCs from the basal cochlea, the stereocilia of IHCs (Supplemental Figure 4, A–D) and OHCs (Supplemental Figure 4, A, B, and E) in the apical region appeared normal. Together, these data identify cellular defects that underpin the hearing loss in *Atf6*^–/–^ mice at 2 months of age: (a) damage to stereocilia on hair cells and (b) reduction of OHC numbers focally.

### Functional pathway analysis identifies enrichment of ER stress, channel activity, actin filament organization, and neuronal death pathways in Atf6^–/–^ cochleae.

**Figure 5:**
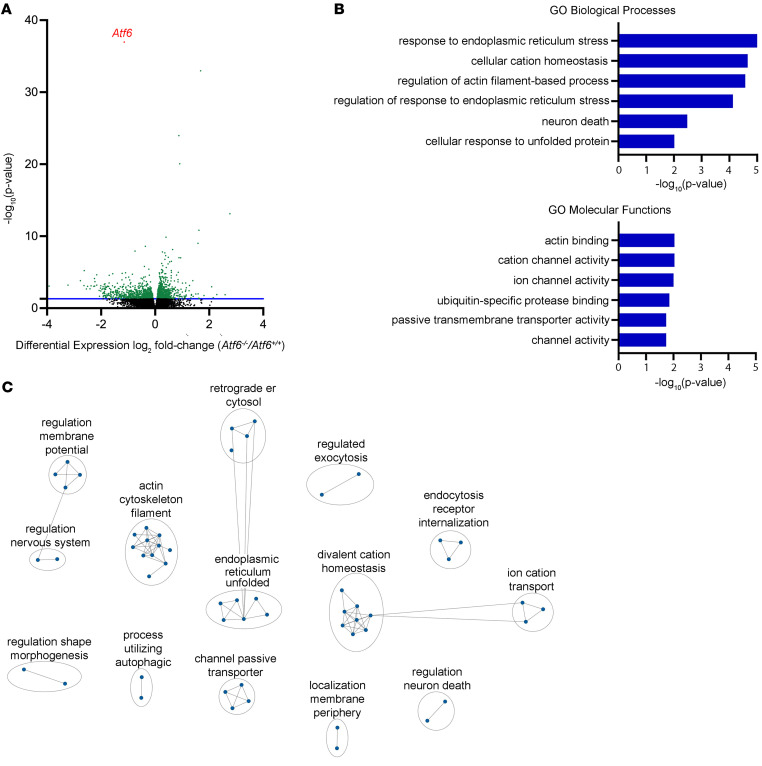
Gene expression in cochleae from 2-month-old *Atf6^+/+^* and *Atf6^–/–^* mice. (**A**) Volcano plot comparing transcript abundance between *Atf6^+/+^* and *Atf6^–/–^* cochleae. The number of DEGs is shown in green (*n* = 1,869), with the statistically significant cutoff (*P* < 0.05 and FC >0.05, above blue line). *Atf6* is indicated in red. (**B**) Go analysis of 1,869 DEGs via g:Profiler revealed changes in ER stress, ion channel regulation, cell death, and actin filament organization. The *y* axis shows the –log_10_ *P* value for the specified term. (**C**) Cytoscape network highlights key enriched themes in the *Atf6^–/–^* mouse cochlear transcriptome.

To identify molecular defects in the cochlea arising from *Atf6* deletion, we analyzed bulk RNA-Seq transcriptomes from *Atf6*^–/–^ and *Atf6*^+/+^ mice cochleae. On the basis of a cutoff for a background value at 0.1 fragments per kilobase of exon per million mapped reads (FPKM), 15,952 transcripts were expressed in *Atf6*^+/+^ and *Atf6*^–/–^ cochleae (Supplemental Table 2). *Atf6^–/–^* mice have slow progressive hearing loss (that resembles the progressive hearing loss reported by our patients carrying ATF6 disease alleles, Figures 1 and 2). Therefore, we adopted a low fold-change (FC >0.05) threshold while maintaining statistical significance in log_2_(FCs) between the 2 groups (Figure 5A, *P* ≤ 0.05 and FC >0.05), yielding 1,869 statistically significant differentially expressed genes (DEGs), represented by green dots above the blue line in Figure 5A.

To gain insight into the functions of the 1,869 genes differentially expressed between *Atf6*^+/+^ and *Atf6*^–/–^ cochlea, we performed gene ontology (GO) analysis (g:Profiler; https://biit.cs.ut.ee/gprofiler/, last accessed October 12, 2022; Supplemental Table 3) (60). Changes in biological processes and molecular function terms (61) were assessed from differential expression of the DEG sets (Figure 5B). GO biological processes analysis revealed that genes differentially expressed between *Atf6^+/+^* and *Atf6^–/–^* cochleae were strongly associated with ER stress (GO:0034976, response to ER stress; GO:1905897, “regulation of response to ER stress”; GO:0034620, “cellular response to unfolded protein”); cellular ion homeostasis (GO:0030003, “cellular cation homeostasis”), actin function (GO:0032970, “regulation of actin filament–based process”), and neuron death (GO:0070997, “neuron death”) (Figure 5B). GO molecular function pathway analysis revealed that DEGs were also strongly associated with actin binding (GO:0003779, “actin binding”), channel activity (GO:0005261, “cation channel activity”; GO:0005216, “ion channel activity”; GO:0022803, “passive transmembrane transporter activity”; GO:0015267, “channel activity”), and ubiquitin-specific protease binding (GO:1990381, “ubiquitin-specific protease binding”) (Figure 5B). Next, Cytoscape (3.8.2) (62) was used to generate an enrichment map of common cellular themes between the biological processes and molecular function GO terms enriched in the *Atf6*^–/–^ cochlea (Figure 5C and Supplemental Figure 5). The enrichment map further highlighted that DEGs were associated with ER protein misfolding (ER unfolded and retrograde ER cytosol nodes), actin cytoskeleton filament, ion homeostasis (divalent cation homeostasis and ion cation transport nodes), and regulation neuron death (Figure 5C). Together, the functional pathway analysis of 2-month-old *Atf6*^–/–^ mouse cochlear transcriptomes revealed notable enrichment of ER stress, channel activity, actin filament organization, and neuron death processes.

### Induction of UPR, ERAD, and ER stress–induced apoptosis pathways in Atf6^–/–^ cochleae.

**Figure 6:**
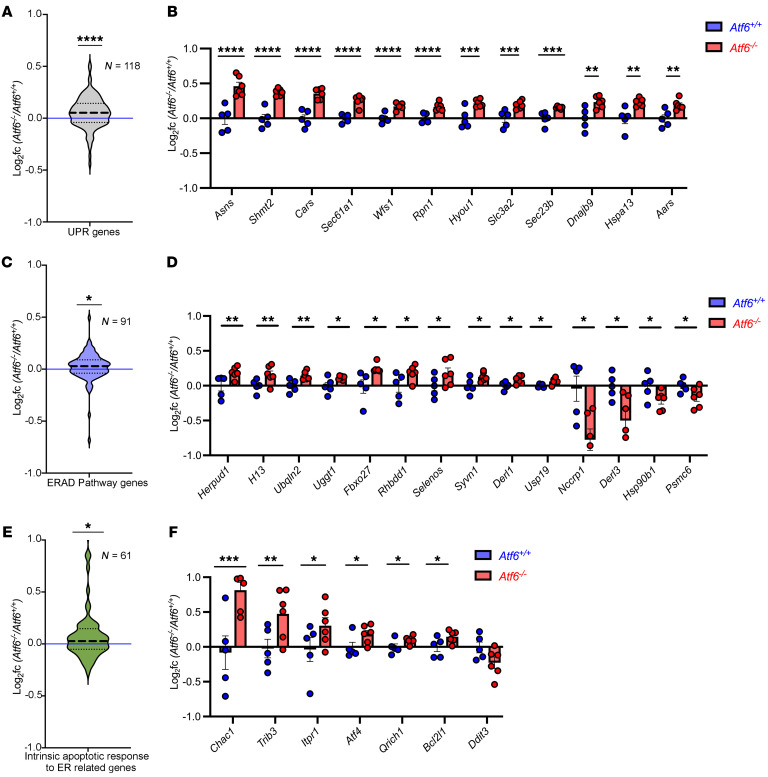
Upregulation of ER stress genes in *Atf6^–/–^* cochlea. (**A**) Gray violin plot shows log_2_(FCs) for 118 UPR-related genes in *Atf6^–^/^–^* versus *Atf6^+/+^* cochleae (****P ≤ 0.0001, Wilcoxon signed-rank test; see also Supplemental Table 4). (**B**) Statistically significant UPR gene expression changes in *Atf6^–^/^–^* (red) versus *Atf6^+^/^+^* (blue) cochleae, with data presented as the mean ± SEM (**P ≤ 0.01, ***P ≤ 0.001, and ****P ≤ 0.0001, DESeq2 analysis). (**C**) Blue violin plot shows log_2_(FCs) for 91 ERAD genes (GO:0036503, “ERAD pathway”) in *Atf6^–/–^* cochleae (*P ≤ 0.05, Wilcoxon signed-rank test; see also Supplemental Table 5). (**D**) Fourteen ERAD genes show statistically significant changes. Data represent the mean ± SEM (*P ≤ 0.05 and **P ≤ 0.01, DESeq2 analysis; see also Supplemental Table 5). (**E**) Green violin plot shows log_2_(FCs) of 61 intrinsic apoptotic genes (GO:0070059, “intrinsic apoptosis response to ER stress”) in Atf6^–/–^ cochleae (*P ≤ 0.05, Wilcoxon signed-rank test; see also Supplemental Table 6). (**F**) Seven apoptotic genes show statistically significant expression changes. Data represent the mean ± SEM (*P ≤ 0.05 and ***P ≤ 0.001, DESeq2 analysis; see also Supplemental Table 6). Dashed lines in the violin plots represent the median and quartiles; the blue horizontal line indicates log_2_ fold = 0 (no change).

ER stress was one of the most enriched processes in the *Atf6*^–/–^ cochlear transcriptome (Figure 5). ER stress triggers many cellular programs including the UPR, ERAD, autophagy, lipid metabolism and synthesis, oxidative phosphorylation, and cell death (2, 63–67). We investigated whether these ER stress–regulated programs were altered in *Atf6*^–/–^ cochlear transcriptomic datasets. In mammals, the UPR is comprised of 3 distinct signal transduction pathways regulated by inositol-requiring enzyme 1 (IRE1), ATF6, and protein kinase RNA-like endoplasmic reticulum kinase (PERK) that generate the XBP1s, ATF6’s cytosolic domain (ATF6f), and ATF4 transcription factors upon ER stress (2). Target genes of XBP1s, ATF6f, and ATF4 have been extensively characterized in mammals (7, 15, 68–74). To determine whether the UPR was induced in the *Atf6*^–/–^cochlea, we probed the expression of a panel of 118-IRE1/XBP1s, ATF6, and PERK/ATF4-regulated target genes (UPR-regulated genes, Supplemental Table 4). We found statistically significant upregulation of the overall UPR-regulated gene panel in *Atf6*^–/–^ cochleae (Figure 6A, gray violin plot, ****P ≤ 0.0001, 2-tailed Wilcoxon signed-rank test). Within the UPR-regulated 118-gene panel, the following 36 genes were statistically significantly altered between *Atf6^–/–^* and *Atf6*^+/+^ cochlea: *Sec61a1*, *Cars*, *Shmt2*, *Asns*, *Wfs1*, *Rpn1*, *Hyou1*, *Slc3a2*, *Sec23b*, *Dnajb9*, *Aars*, *Hspa13*, *Tmem50b*, *Sec24d*, *Vegfa*, *Herpud1*, *Slc2a6*, *Ficd*, *Txndc11*, *Cbx4*, *Slc1a4*, *Rbm10*, *Sec31a*, *Piga*, *Trib3*, *Atf4*, *Syvn1*, *Hsp90b1*, *Derl1*, *Cnnm2*, *Derl3*, *Pdia4*, *Nucb2*, *Nars*, *Arfgap3*, and *Lman1* (Figure 6B, Supplemental Figure 6, and Supplemental Table 4; ****P ≤ 0.0001, ***P ≤ 0.001, **P ≤ 0.01, *P ≤ 0.05, by DESeq2 analysis, Supplemental Table 4). Interestingly, nearly all (32 of 36 genes) notably altered UPR genes were upregulated in *Atf6*^–/–^ cochleae, and the top 12 most strongly induced UPR genes were closely linked to the PERK/ATF4 pathway (69–71) (Figure 6B). By contrast, all 4 statistically significantly downregulated genes in *Atf6*^–/–^ cochleae (*Tmem50b*, *Slc2a6*, *Hsp90b1*, and *Derl3*) were linked to the ATF6 pathway (Supplemental Figure 6 and Supplemental Table 4) (7, 15, 68). Thus, our findings provide evidence that the UPR, especially the PERK arm, is notably activated by ER stress in *Atf6*^–/–^ cochleae. Furthermore, gene set enrichment analysis (GSEA) also confirmed marked enrichment in related terms: “response to ER stress” (FDR = 0.18) and “cellular response to unfolded protein” (FDR = 0.18) in the *Atf6^–/–^* cochlear transcriptome (Supplemental Figure 7).

ERAD is another cellular mechanism triggered by ER stress (63, 66, 72, 75). In our GO analysis of the *Atf6*^–/–^ cochlear transcriptome, we did not find a statistically significant association of the GO ERAD term (GO:0036503), but we did observe a statistically significant association of a related GO term, ubiquitin-specific protease binding (Figure 5). To investigate ERAD status in *Atf6*^–/–^ cochlea in more detail, we queried expression levels of 91 genes in the ERAD pathway gene set (Supplemental Table 5). We found a statistically significant increase in median expression of the ERAD panel in *Atf6*^–/–^ versus *Atf6*^+/+^ cochleae (Figure 6C, violin plot, *P ≤ 0.05, 2-tailed Wilcoxon signed-rank test). Within the gene panel, 14 ERAD pathway genes (*Herpud1*, *H13*, *Ubqln2*, *Uggt1*, *Fbxo27*, *Rhbdd1*, *Selenos*, *Syvn1*, *Derl1, Usp19*, *Nccrp1*, *Derl3*, *Hsp90b1*, and *Psmc6*) were statistically significantly different in *Atf6*^–/–^ versus *Atf6*^+/+^ cochleae, and 10 of these 14 ERAD genes were upregulated in *Atf6*^–/–^ mouse cochleae (Figure 6D, *P ≤ 0.05, **P ≤ 0.01, ****P ≤ 0.0001, by DESeq2 analysis, Supplemental Table 5). Thus, these results support the idea that ERAD is another ER stress–regulated mechanism induced in *Atf6*^–/–^ mouse cochlea.

Excessive or chronic ER stress triggers cell death (76–78). We saw hair cell dropout in *Atf6*^–/–^ cochleae (Figure 4), and our GO analysis also revealed that the DEGs were notably associated with the cell death term “neuron death” in *Atf6*^–/–^ cochleae (Figure 5). To investigate in more detail if ER stress–related cell death was induced in *Atf6*^–/–^ cochlea, we queried expression levels of the 61 genes associated with the GO term “intrinsic apoptotic response to ER stress” (GO:0070059) in *Atf6*^–/–^ cochlear transcriptomes (Supplemental Table 6). We observed a statistically significant increase in the median expression of the intrinsic apoptotic response to the ER stress gene panel in *Atf6*^–/–^ versus *Atf6*^+/+^ cochleae (Figure 6E, violin plot, *P ≤ 0.05, 2-tailed Wilcoxon signed-rank test). Within the 61-gene ER stress apoptotic response panel, 6 genes (*Chac1*, *Trib3*, *Itpr1*, *Atf4*, *Qrich1*, and *Bcl2l1*) were statistically significantly changed in *Atf6*^–/–^ versus *Atf6*^+/+^ cochlear transcriptomes, and these were all upregulated in *Atf6*^–/–^ mice (Figure 6F, *P ≤ 0.05, **P ≤ 0.01, ***P ≤ 0.001, by DESeq2 analysis, Supplemental Table 6). Together, these results show that UPR, ERAD, and ER stress–induced cell death were notably induced in the *Atf6^−/−^* cochlear transcriptome. By contrast, autophagy, oxidative stress, and lipid synthesis/metabolism were not statistically significantly altered in the *Atf6*^–/–^ cochlear transcriptome (Supplemental Figure 8 and Supplemental Table 7).

### DEG analysis identifies dysregulation of actin filament–/stereocilia-related and channel-related genes in Atf6^–/–^ cochleae.

**Figure 7:**
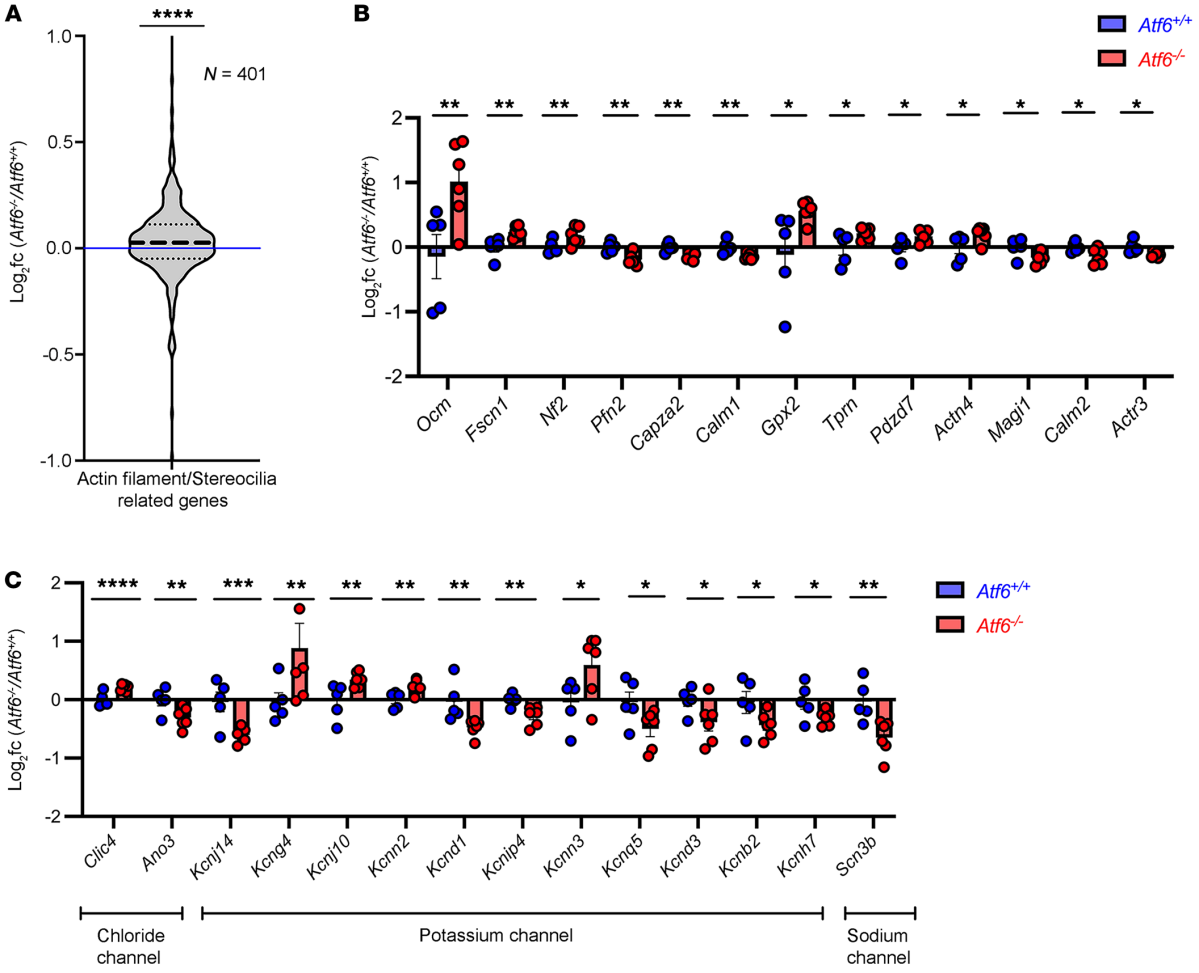
Dysregulated actin filament–/stereocilia- and channel-related gene expression in *Atf6^–/–^* cochleae. (**A**) Violin plots (gray) show log_2_(FCs) in 401 actin filament–/stereocilia-related genes in *Atf6^–/–^* versus *Atf6^+/+^* cochlear transcriptomes (****P ≤ 0.0001, 2-tailed Wilcoxon signed-rank test; see also Supplemental Table 8). The thick dashed line indicates the median, the thin dashed lines show quartiles, and the blue line indicates no FC. (**B**) Thirteen stereocilia genes showed statistically significant changes between *Atf6^–/–^* (red) and *Atf6^+/+^* (blue) cochleae. Data indicate the mean ± SEM (*P ≤ 0.05 and **P ≤ 0.01, DESeq2 analysis; see also Supplemental Table 8). (**C**) Statistically significant changes in gene expression for chloride, potassium, and sodium channels in *Atf6^–/–^* versus *Atf6^+/+^* cochleae are shown, with individual cochleae represented as circles. Data are presented as the mean ± SEM (*P ≤ 0.05, **P ≤ 0.01, ***P ≤ 0.001, and ****P ≤ 0.0001, DESeq2 analysis; see also Supplemental Table 9).

GO analysis of *Atf6*^–/–^ cochlear transcriptomes also revealed a notable association with terms related to actin filament organization (Figure 5, B and C). GSEA confirmed enrichment in related terms: “actin filament bundle assembly” (FDR = 0.11) and “auditory receptor cell stereocilium organization” (FDR = 0.14) in the *Atf6^–/–^* cochlear transcriptome (Supplemental Figure 9). Hair cell stereocilia consist of bundles of highly crosslinked actin filaments (F-actin) (79), and hair cell stereocilia were extensively damaged in hair cells of *Atf6*^–/–^ mice (Figure 4). To investigate in more detail how the loss of *Atf6* in cochlea affected actin-rich stereocilia genes, we queried combined actin filament–/stereocilia-related gene sets (actin filament gene set: GO:0007015; stereocilia gene set is given in refs. 80, 81) (Figure 7A and Supplemental Table 8). We found that the overall mean expression of the 401 actin filament–/stereocilia-related combined gene panel was statistically significantly increased in *Atf6*^–/–^ cochlear transcriptomes (Figure 7A, violin plot, ****P ≤ 0.0001, 2-tailed Wilcoxon signed-rank test). Furthermore, analysis of 105 stereocilia-enriched genes within the 401-gene panel (80, 81) showed a statistically significant increase in *Atf6*^–/–^ cochlear transcriptomes (Supplemental Figure 10, violin plot, *P ≤ 0.05, 2-tailed Wilcoxon signed-rank test, and Supplemental Table 8) and identified 13 stereocilia genes (*Ocm*, *Fscn1*, *Nf2*, *Pfn2*, *Capza2*, *Calm1*, *Gpx2*, *Tprn*, *Pdzd7*, *Actn4*, *Magi1*, *Calm2*, and *Actr3*) that were statistically significantly different between *Atf6*^–/–^ and *Atf6*^+/+^ mice (Figure 7B, *P ≤ 0.05, **P ≤ 0.01, by DESeq2 analysis, Supplemental Table 8). Thus, our analysis showed dysregulated expression of actin filament–/stereocilia-related genes in *Atf6*^–/–^ cochlea that could correlate with or directly underlie the disorganization of hair cell bundles seen by microscopy.

Last, we investigated the expression of ion channel–related genes in *Atf6*^–/–^ versus *Atf6*^+/+^ cochlear transcriptomes because GO analysis identified multiple channel terms enriched in the *Atf6*^–/–^ cochlear transcriptome (Figure 5B). We saw no changes in the mean expression levels of channel-related gene sets but did find statistically significant alterations of several individual genes encoding chloride channel genes (*Clic4* and *Ano3*), potassium channel genes (*Kcnj14*, *Kcng4*, *Kcnj10*, *Kcnn2*, *Kcnd1*, *Kcnip4*, *Kcnn3*, *Kcnq5*, *Kcnd3*, *Kcnb2*, and *Kcnh7*), and a sodium channel gene (*Scn3b*) in *Atf6*^–/–^ versus *Atf6*^+/+^ cochleae (Figure 7C, *P ≤ 0.05, **P ≤ 0.01, **P ≤ 0.001, ****P ≤ 0.0001, Supplemental Table 9) (80). By contrast, calcium channel–related genes were not statistically significantly altered between *Atf6*^–/–^ and *Atf6*^+/+^ cochleae in cochlear transcriptomes (e.g., *Cacnb1*, *Cacnb2*, and *Cacnb3*). Taken together, a subset of these ion channel genes also showed dysregulation (both up- and downregulated expression) in *Atf6*^–/–^ cochlea.

## Discussion

ATF6 controls a key signal transduction pathway of the UPR that helps cells adapt to ER stress. In people, cone photoreceptors require ATF6 for development and function, and loss of ATF6 leads to congenital vision loss diseases like achromatopsia (10, 13, 15, 67). Here, we report that hair cells in the OC also require ATF6 for function and viability and that loss of ATF6 leads to a second human disease — sensorineural hearing loss, which is phenocopied in *Atf6^–/–^* mice. Cochleae from these mice showed extensive damage to hair cell stereocilia and focal hair cell loss. Transcriptional analysis of cochleae from *Atf6^–/–^* mice revealed a marked induction of the UPR transcriptional program, especially through the PERK arm. These current findings, coupled with our previous studies, indicate that ATF6 inactivation causes a vision and hearing loss syndrome in people arising from cone photoreceptor and hair cell dysfunction. Furthermore, at the molecular level, hair cell damage and hearing loss arising from the loss of ATF6 were linked to ER stress and UPR activation in the cochlea.

We visualized hair cell damage in cochleae of 2-month-old *Atf6^–/–^* mice with hearing loss (Figures 2 and 4), and the patient audiometric testing results showed that human hair cells were also defective when ATF6 function was lost. Otoacoustic emissions, which are sounds generated by healthy OHCs in the cochlea (82, 83), were absent in 2 patients. Despite detailed clinical evaluations, 1 patient showed limited responses at 3 kHz in the right ear and at 2 kHz in the left ear. A heightened response at 5 kHz in the left ear likely resulted from reduced olivocochlear suppression, causing increased cochlear gain (84). The results indicate that there was loss of OHCs in the patient with ATF6 loss. Furthermore, audiograms in these patients showed downward slopes at high frequencies (Figure 1), a pattern typically linked to OHC defects (85, 86). Similar to the quality of the auditory recording defects found in patients, the 2-month-old *Atf6^–/–^* mice also showed statistically significantly higher ABR thresholds with high-frequency stimuli (32 kHz) compared with lower-frequency stimuli (Figure 2B). Thus, these clinical auditory phenotypes support a common pathogenic mechanism causing hearing loss in humans and mice with loss of ATF6 function. Disease-associated ATF6 variants found in humans disrupt transcriptional signaling via distinct pathomechanisms (11). Further investigation of audiograms from patients across a broader range of ATF6 variants is needed to determine the role of ATF6 in sensorineural hearing loss and whether auditory phenotypic differences exist between ATF6 genotypes.

The 2-month-old *Atf6^–/–^* mice showed markedly increased ABR thresholds at all frequencies tested (Figure 2) that corresponded, anatomically, with apical (low frequency) to basal (high frequency) increasing cochlear pathology. *Atf6^–/–^* mice showed stereocilia disorganization of IHCs and OHCs in the basal cochlea, with hair cell loss limited to OHCs in this region (Figure 4). Prior mouse experiments have demonstrated that the basal cochlea in mice is most susceptible to noise and ototoxic drugs (87–89). Hu et al. found that OHC death starts in the basal turns and spreads apically in Cdh23erl/erl cochleae (87). Ikaheimo et al. reported stereocilia fusion and synapse degradation in basal OHCs and IHCs of Manf-deficient mice (90). Fujinami et al. showed that ER stress impairs high-frequency hearing in tunicamycin-treated rats due to basal OHC damage (91). Jongkamonwiwat et al. revealed that high-frequency regions are prone to noise-induced stereocilia damage (58). On the basis of these findings, we propose that *Atf6* deficiency confers susceptibility to ER stress throughout the cochlea. According to this model, the genetic vulnerability to ER stress, compounded with natural environmental ER stressors encountered by the cochlea during life, causes OHCs to begin to drop out in the basal region. Then, hair cell loss spreads to the rest of the cochlea, accounting for the deterioration of hearing in ATF6 patients and mice.

What are possible mechanisms that make hair cells vulnerable to the loss of ATF6? Hair cells require numerous membrane and structural proteins to maintain the stereocilia’s hair-like structure, essential for sound detection. Without ATF6, stereocilia may disintegrate, as their necessary proteins are not produced in sufficient quantity or quality. Similarly, we previously found that photoreceptors, which also have polarized cilia–derived structures (the photoreceptor outer segments), are highly sensitive to ATF6 loss (10, 12, 15). These findings suggest that ATF6 is crucial for the development, stability, and function of polarized sensory neurons.

Another mechanism that could explain hair cell vulnerability to ATF6 loss relates to hair cells’ susceptibility to noise-induced proteotoxicity. Excessive noise disrupts proteostasis in hair cells and is a primary cause of progressive hearing loss in people (58). Proteomics and transcriptional analyses of noise-exposed mouse cochleae show increased chaperone and degradation activity, as well as activation of the UPR and ERAD to combat noise-induced protein damage (58). In addition to noise, ER stress–inducing chemicals or aging can also lead to ER stress and hearing loss in animal and in vitro cell assays (91–96). These findings highlight noise as a major cause of cochlear ER stress and help explain why ATF6 is essential for preserving cochlear health and hearing.

Highlighting the importance of protein quality control to cochlear health, sensorineural hearing loss is a frequent phenotype when ER quality control genes are mutated. Similar to *Atf6^–/–^* mice, mice lacking the ER quality control regulator mesencephalic astrocyte-derived neurotrophic factor (*MANF*), show hair cell stereocilia disarray, progressive OHC death, elevated ABR thresholds, and UPR induction in the cochlea (88, 90). Clinical audiograms from a patient with a homozygous loss-of-function *MANF* variant also revealed severe bilateral sensorineural hearing loss that progressively worsened with age (90). However, early-onset juvenile diabetes arises with the loss of MANF in mice and humans (97), but not in *Atf6^–/–^* mice, and has not been reported in patients carrying ATF6 loss-of-function variants. In people, disease variants of Wolfram syndrome 1 (*WFS1*), another ER quality control component, cause autosomal recessive sensorineural hearing loss (98), and mice homozygous for exon 8 deletion or an E864K missense change of WFS1 also develop hearing defects (99). WFS1-associated deafness is also linked with diabetes insipidus, diabetes mellitus, and retinal ganglion cell–related optic nerve atrophy in people as part of the clinical symptoms of Wolfram syndrome and DIDMOAD (diabetes insipidus, diabetes mellitus, optic atrophy, and deafness) (100, 101). In mice, genetic inactivation of transmembrane and tetratricopeptide repeat 4/transmembrane O-mannosyltransferase targeting cadherins 4 (*Tmtc4*), another ER quality control regulator, also triggers hearing loss, hair cell death, and UPR activation (102), reminiscent of the defects observed in *Atf6^–/–^* mice. These studies support that proteostasis genes and mechanisms are critical for cochlear health and function.

Our current study shows that ATF6 is essential for hearing, and our prior studies showed that ATF6 is essential for vision in people (10, 13, 15, 67). This combination of auditory and visual dysfunction, along with the biallelic inheritance of *ATF6* variants, mirrors Usher syndrome, characterized by blindness, deafness, and autosomal recessive inheritance (103). Many mouse models of Usher syndrome recapitulate the auditory defects found in patients but show little to no vision defects (103), and *Atf6^–/–^* mice also exhibit hearing loss (Figures 1, 2, and 4) but retain normal vision until 18 months of age, when a gradual decline in photopic and scotopic responses occurs (10, 19). At the subcellular level, Usher syndrome genes and ATF6 share no obvious mechanistic functions. Many Usher syndrome proteins are structural components of stereocilia in hair cells and ciliary/periciliary/calyceal processes in photoreceptors, whereas ATF6 is an ER membrane–bound transcription factor that regulates ER protein-folding fidelity. Therefore, we propose that ATF6 inactivation causes a syndrome in patients with phenotypic (blindness-deafness) and cellular (photoreceptor–hair cell) features that resemble Usher syndrome, combined with molecular pathomechanisms (ER stress, UPR dysregulation) that are causal in Wolfram syndrome.

Sensorineural hearing loss is one of the most common sensory disorders affecting approximately 466 million people worldwide (104). Small molecules that modulate protein quality control offer promising therapeutic strategies for protection against sensorineural hearing loss by maintaining cochlear protein quality (87, 88, 90–96, 102, 105). For example, the chemical chaperones taurine-conjugated derivative of ursodeoxycholic acid (TUDCA), preserved hair cells and delayed hearing loss in *erl* mice (106). Small-molecule inhibition of the PERK arm of the UPR and integrated stress response (ISR) using integrated stress response inhibitor (ISRIB) protected hair cells and attenuated hearing loss in the *Tmtc4^–/–^* mouse (102) as well as in a rodent model of noise-induced hearing loss (107). Beyond PERK, our study suggests that targeting the ATF6 and IRE1 arms of the UPR may also benefit cochlear health. Human and mouse genetic data indicate that ATF6 inactivation contributes to hearing loss, making small-molecule ATF6 activators a potential therapy. AA147, which enhances ATF6 activity, is effective in restoring ATF6 function in retinal organoids from patients with ATF6 disease variants (15, 108). In addition, the XBP1s transcription factor, generated by the IRE1 arm of the UPR, shares many gene targets with ATF6 (15, 75, 109). Therefore, small-molecule IRE1-XBP1s activators may also have otoprotective properties. Conversely, small-molecule ATF6 inhibitors (e.g., CeapinA7) and IRE1-XBP1 inhibitors may have ototoxic side effects. Our study highlights both the ototoxic effects of ER stress dysregulation and the potential for drugs to mitigate ER stress–related ototoxicity.

## Methods

### Sex as a biological variable.

All studies included a minimum of 2 animals of each sex per group, and no sex-specific effects were found.

### Patients.

The family carrying the point mutation (c.970C>T, p.Arg324Cys) and a patient with the point mutation (c.1699T>A, p.Tyr567Asn) underwent comprehensive otologic and audiometric evaluations through their health care providers. Otoscopy revealed no external ear abnormalities in any of the patients. Audiometric examinations, conducted by registered audiologists, measured air conduction pure-tone hearing thresholds at 0.25, 0.5, 1, 2, 3, 4, 6, and 8 kHz in each ear.

### Animals.

Transgenic *Atf6^+/+^* and *Atf6^–/–^* (7, 10, 19) mice on a pure C57BL/6J (B6J) background were originally developed by Randal Kaufman (Sanford Burnham Prebys Medical Discovery Institute, La Jolla, California, USA) and were used and maintained in our laboratory (Stanford University, Stanford, California, USA). The B6J strain has been extensively used as a model for early-onset, age-related hearing loss (33–36). B6J mice show high-frequency hearing loss that begins at 3–6 months of age and which then progresses in severity and spreads to lower frequencies with advancing age over the next 12–15 months (37). All experiments used female or male *Atf6^–/–^* mice and their control *Atf6^+/+^* littermates at P14 (i.e., 2 weeks, *n* = 8 *Atf6^+/+^*; *n* = 8 *Atf6^–/–^*) and P60 (i.e., 2 months, *n* = 10 *Atf6^+/+^*; *n* = 6 *Atf6^–/–^*). For all experiments, animals were kept in cyclic 12-hour light/12-hour dark conditions with free access to food and water.

### Auditory brainstem response.

The protocols for ABR acquisition in mice were performed as previously published in detail (110). All acquisitions were performed in a sound-attenuating chamber (Sonora Technology Co.). Prior to input in the chamber, mice were anesthetized using a combination of ketamine (40 mg/kg; KETASET) and xylazine (8 mg/kg; X-Ject SA, Butler) using procedures similar to those outlined in our published protocols (110). Following deep anesthesia, the animals were maintained on a warming pad, and 3 needle electrodes (recording, reference, and ground) were inserted subcutaneously. Tone pips (3 ms duration, frequencies at 8, 12, 16, 24, 28, and 32 kHz, and intensities at 20, 40, 60, or 80 dB SPL) were delivered to the left ears at a rate of 19 times per second through a calibrated earphone (Stax Ltd.). ABR signals were recorded using BioSigRP software on a Tucker Davis Technology System 3 recording rig (Tucker-Davis Technologies), and 512 recordings were averaged for each animal in each condition. Following ABR testing, the same cohort of mice underwent comprehensive histological examination of both the middle and IE tissues.

### Tissue preparation.

Mice were anesthetized using a combination of ketamine (40 mg/kg; KETASET) and xylazine (8 mg/kg; X-Ject SA, Butler) at P14 and P60. IEs and MEs were dissected and fixed in 4% paraformaldehyde overnight at 4°C. IEs and MEs were later decalcified in 0.5 M EDTA, pH 7.5, overnight at 4°C (Merck) or until clear and later placed in 30% sucrose for cryoprotection at 4°C until fully sunken. After decalcification, cochleae were mounted in OCT compound for cryostat sectioning. Cochlear sections of 10 μm thickness were collected with a Leica CM1950 cryostat (Leica Biosystems) and placed on Superfrost Plus slides (Thermo Fisher Scientific). For whole mounts, the OC was microdissected as previously described (55).

### H&E staining.

Cochlear sections (10 μm) were collected on gelatin-coated slides for H&E staining. Slides were dipped in Harris hematoxylin for 1 minute and then washed in tap water and dehydrated in alcohol. Slides were then dipped in eosin-phloxyine for 30 seconds and then dehydrated in a series of 95% ethanol and 100% ethanol, followed by 5 minutes in xylene and mounting in xylene-based mounting medium (Vector Laboratories). ImageJ software, version 1.50i (NIH) was used to measure the thickness of the SV. For each section, 3 measurements of the SV thickness were taken at each section, spaced approximately 50 μm apart, and the measurements were then averaged for each section. SV thickness measurements were collected for 6 cochleae from individual 2-month-old *Atf6^+/+^* and *Atf6^–/–^* mice. In addition, the number of SG cells in both *Atf6^+/+^* and *Atf6^–/–^* mouse cochleae were manually counted at 2 months (*n* = 6).

### Cochlear whole mounts and IHC.

For whole-mount IHC, cochleae were fixed in 4% PFA overnight at 4°C and then decalcified with 0.5 M EDTA overnight at 4°C. The OC was microdissected as above. Whole mounts were washed with PBS and then incubated with 0.05% Triton in 10% normal donkey serum (NDS) for 1 hour at room temperature. Whole mounts were then incubated overnight at 4°C with rabbit anti–myosin VI primary antibody (catalog 25-6790, Proteus Biosciences) at a dilution of 1:300 in PBS. The whole mounts were then washed with PBS and incubated for 4 hours at room temperature with the secondary antibody donkey anti–rabbit IgG Alexa Flour 488 (catalog A-21206, 1:300 dilution, Thermo Fisher Scientific) and Texas Red-X–conjugated phalloidin (catalog T7471, 1,400 dilution, Thermo Fisher Scientific). Whole mounts were again washed with PBS and mounted with Vectashield Antifade Mounting Media (catalog H-1000-10, Vector Laboratories). Images were collected with a Leica SP8 confocal microscope with lightning deconvolution and processed using LasX Image Analysis software at the UCSD School of Medicine Microscopy Core.

### RNA-Seq analysis.

RNA-Seq analysis was performed as previously described (15). Whole cochleae from mice were collected, and RNA extraction was performed following the manufacturer’s instructions (catalog 74104, QIAGEN RNAeasy Mini kit). RNA-Seq was performed by the Eukaryotic Strand-specific Transcriptome Resequencing service of BGI (http://biosys.bgi.com) using the proprietary DNBSEQ stranded mRNA library and with paired-end 100 bp reads at 30 million reads per sample. Alignment of the sequencing data was performed using the HISAT2 alignment program (version 2.0.4) to the Mus_musculus_GCF_000001635.27_GRCm39. Gene expression and normalized reads (in FPKM) were determined with RSEM software (version 1.2.18) (111). The DESeq2 package (version 1.4.5) (112) was used to determine differential expression between the control and experimental groups and to calculate the statistical significance of our findings.

### Functional enrichment analysis.

g:Profiler (University of Tartu, Tartu, Estonia; https://biit.cs.ut.ee/gprofiler/) was used for functional enrichment analysis. GO analysis was used for functional annotation and pathway analysis (molecular functions, biological processes) (113). From the RNA-Seq experiments, genes with statistically different log_2_(FC) expression levels (*P* ≤ 0.05, >0.1 FPKM) and a FC of greater than 0.05 between *Atf6^+/+^* and *Atf6^–/–^* cochleae were used as the input. In our study, the *Atf6^–/–^* mice had slow, progressive hearing loss (that resembled the progressive hearing loss reported by our patients carrying ATF6 disease alleles). Therefore, we adopted a low FC of greater than 0.05 threshold but still maintained a statistically significant *P* value of less than 0.05 in defining 1,869 DEGs for pathway analysis of the original 15,000+ genes identified with differential expression between mutant and WT.

These 1,869 DEGs used in our study identified enrichment of ER stress pathways in *Atf6^–/–^* cochleae. This finding is compatible with previous studies of other cell types and tissues in *Atf6^–/–^* mice that also found ER stress dysregulation (7, 8, 19–21) and supports the physiological relevance of the DEGs used in our study.

The gene enrichment map file, containing enriched terms from all databases, produced by g:Profiler was imported into Cytoscape (version 3.8.2, Institute for Systems Biology), and visualization of all terms into enrichment map was done with the Cytoscape plug-in, Enrichment Map (114). Terms were grouped together into clusters by another Cytoscape plug-in, clusterMaker2. The created clusters were labeled by the Cytoscape plug-in Auto Annotate (115, 116). Alternatively, GSEA software (Broad Institute) was used to perform functional enrichment analysis. Preranked lists were entered with the same gene sets and ranked on the basis of expression values relative to WT controls. Weighted analysis with the GO reference database was performed, and GSEA enrichment plots are presented.

### Statistics.

All data are expressed as the mean ± SEM. Statistical significance was defined as a *P* value of less than 0.05. All statistics were calculated using GraphPad Prism 9 (GraphPad Software). A 2-way ANOVA was used to compare average ABR thresholds at 5 frequency regions (8, 12, 16, 24, 28, and 32 kHz) between *Atf6^+/+^* and *Atf6^–/–^* mice (Figure 2). To evaluate whether the thickness of the SV and the number of SG cells/area differed between *Atf6^+/+^* and *Atf6^–/–^* cochleae, measurements were quantified by ImageJ (version 1.53, NIH). The Welch’s *t* test was used to examine the difference between 2 different means (Figure 3, E and F). To evaluate whether the number of hair cells (i.e., OHCs and IHCs) and the number disorganized bundles of stereocilia in IHCs differed between *Atf6^+/+^* and *Atf6^–/–^* cochleae, a Welch’s *t* test was used to examine the difference between 2 different means (Figure 4, B and D). Violin plots comparing *Atf6^+/+^* and *Atf6^–/–^* transcriptomes were generated using the log_2_(FC) data from the differential expression analysis for UPR-related genes, ERAD genes, intrinsic apoptotic response to ER-related genes, and actin filaments/stereocilia-related genes. Differences in expression of gene sets were evaluated for statistical significance using a 2-tailed Wilcoxon signed-rank test (Figure 6, A, C, and E, and Figure 7A). For analyses of RNA-Seq data, statistical significance was calculated for differences in expression of UPR-related genes, ERAD genes, intrinsic apoptotic response to ER-related genes, stereocilia-related genes, chloride channel genes, potassium channel genes, and sodium channel genes and are reported as *P* values for 6 *Atf6^–/–^* and 5 *Atf6^+/+^* individual cochleae calculated by DESeq2 analysis (Figure 6, B, D, and F, Figure 7, B and C, and Supplemental Table 2).

### Study approval.

All mouse procedures were approved by the IACUCs of Stanford University and UCSD. Participants with ACHM provided written informed consent, approved by the Ethics Committee at University College London Hospital and the Research Ethics Board at the University of Toronto and followed the principles of the Declaration of Helsinki.

### Data availability.

RNA-Seq data are available at the NCBI Gene Expression Omnibus (GEO) database (GEO GSE242321). All other data are provided in the article and/or in the Supporting Data Values file.

## Author contributions

EJL and JHL designed all the experiments and interpreted the results. JHL provided ethics oversight and secured funding. Experimental contributions were made by EJL, KK, LAS, and EC. RNA-Seq and analysis were performed by EJL, KK, MSDA, HM, and GZ. Data and statistical analysis were conducted by EJL, KK, GZ, and MSDA. Clinical data collection was carried out by KJS and JHL. The manuscript was written by EJL and JHL. All authors, including AFR, contributed to the review and editing of the manuscript.

## Supplementary Material

Supplemental data

Supplemental table 1

Supplemental table 2

Supplemental table 3

Supplemental table 4

Supplemental table 5

Supplemental table 6

Supplemental table 7

Supplemental table 8

Supplemental table 9

Supporting data values

## Version 1 11/21/2024

In-Press Preview

## Version 2 02/03/2025

Electronic publication
