# Supplementary material for: Mutations in unfolded protein response regulator ATF6 cause hearing and vision loss syndrome
Source: J Clin Invest. 2025 Feb 3;135(3):e175562. doi: 10.1172/JCI175562 (PMC11785932; doi:10.1172/JCI175562)
Supplement: Supplemental data [file jci-135-175562-s164.pdf]

Supplemental Figures and Legends

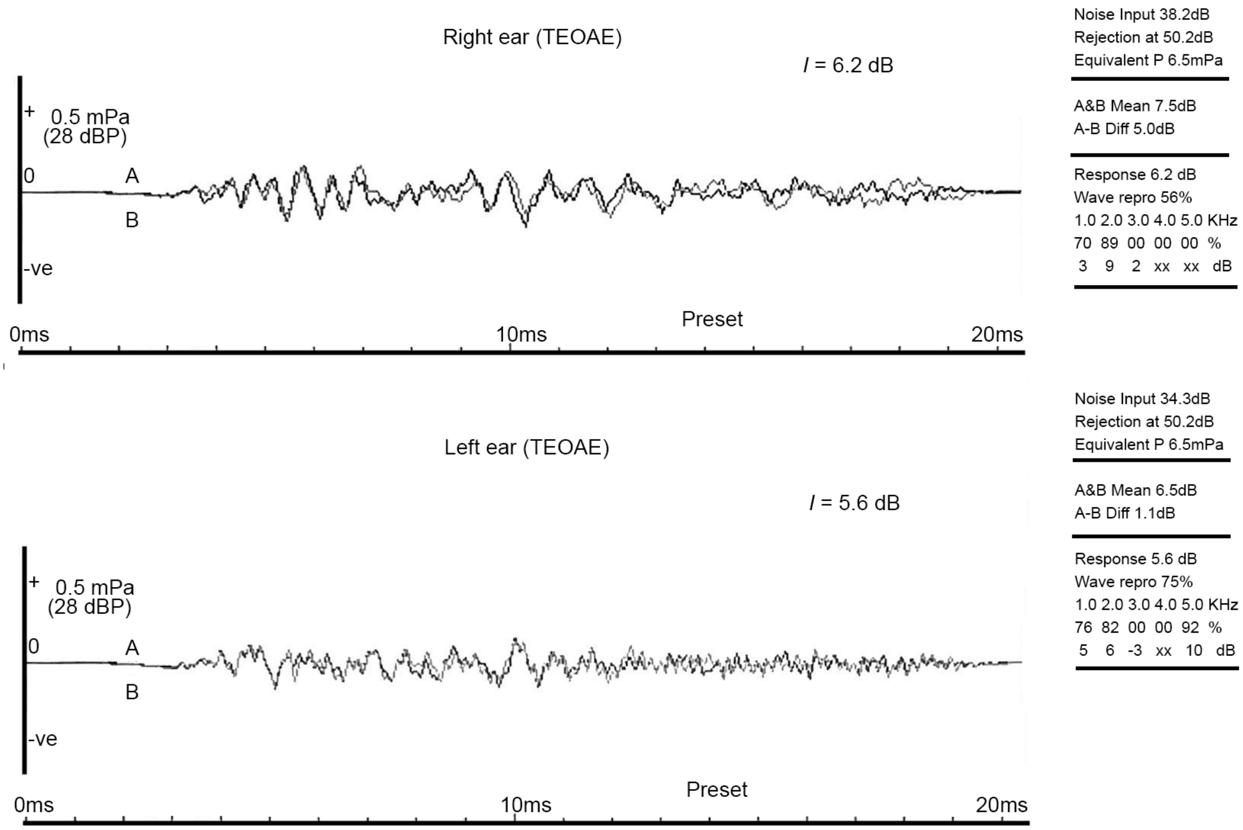

Supplemental Figure 1. Transient evoked otoacoustic emission (TEOAE) are recorded in the right and left ear from II:3 patient carrying homozygous p.Arg324Cys mutations at age 31. The temporal waveforms are shown in the *left panel* (amplitude in millipascals plotted against time in milliseconds), with an analysis time between 2.6 and 20 ms after the stimulus onset (stimulus artifact rejected). 'I' indicates the TEOAE amplitude in decibels SPL. Subject II:3 shows some response but only up to 3KHz on the right and 2KHz on the left, and unusually high response amplitude in the 5KHz band on the left (10 dB SPL). dB, decibel; Hz, hertz; millipascals, mPa.

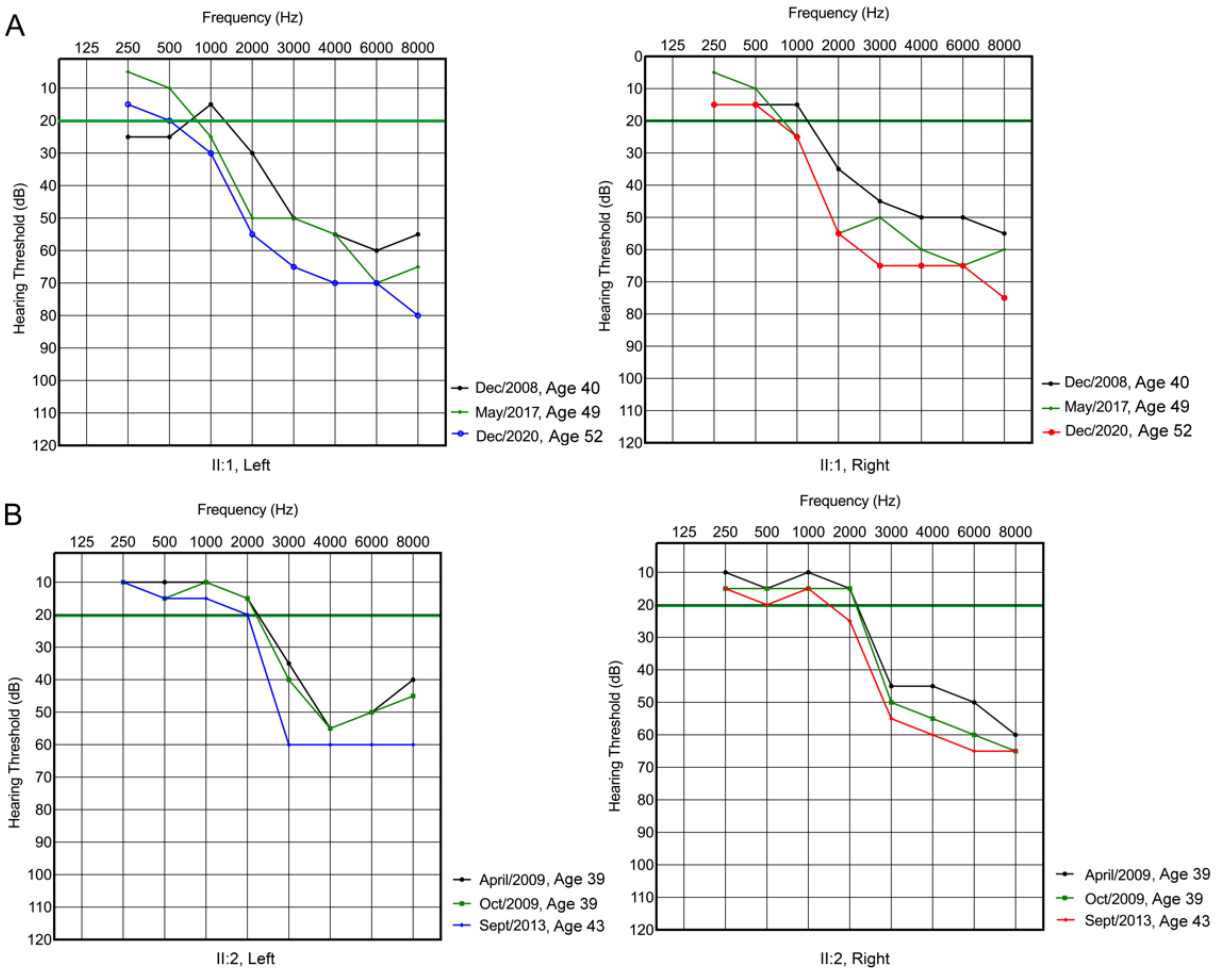

Supplemental Figure 2. Audiograms of both ear affected subjects in family carrying the point mutation (c.970C>T, p.Arg324Cys). (A) Audiograms from II:1 patient carrying homozygous p.Arg324Cys mutations at ages 40, 49, and 52 show progressive hearing loss at high frequencies in left (black, green, and blue lines) and right (black, green, and red lines) ears. (B) Audiograms from II:2 patient carrying homozygous p.Arg324Cys mutations at ages 39 and 43 show progressive hearing loss at high frequencies in left (black, green, and blue lines) and right (black, green, and red lines) ears. Green line at 20 dB indicates normal hearing threshold. dB, decibel; Hz, hertz.

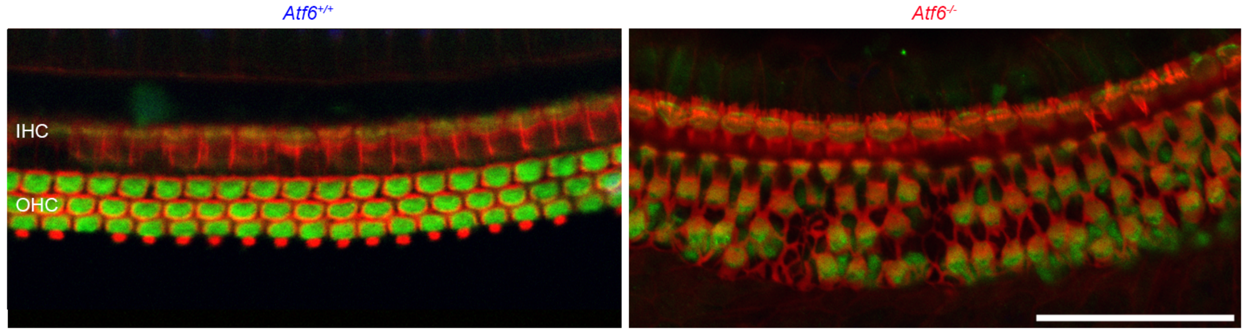

Supplemental Figure 3. Cochlear hair cells of *Atf6*$^{-/-}$ mice exhibit disorganized outer hair cells in the basal region (Approximate frequency 28-32kHz). *Atf6*$^{+/+}$ and *Atf6*$^{-/-}$ cochleae were stained for myosin 7a (MYO7A, green) and phalloidin (red) to visualize hair cells and stereocilia, respectively. Immunofluorescent confocal images of 2-month-old *Atf6*$^{-/-}$ outer hair cells show loss of OHCs and disrupted stereocilia in the IHCs. The image of the *Atf6*$^{-/-}$ cochlea provides a broader view of both OHCs and IHCs, complementing Figures 4A and 4C.Scale bar: 50 μm. OHC, outer hair cell; IHC, inner hair cell. Scale bar, 50 um.

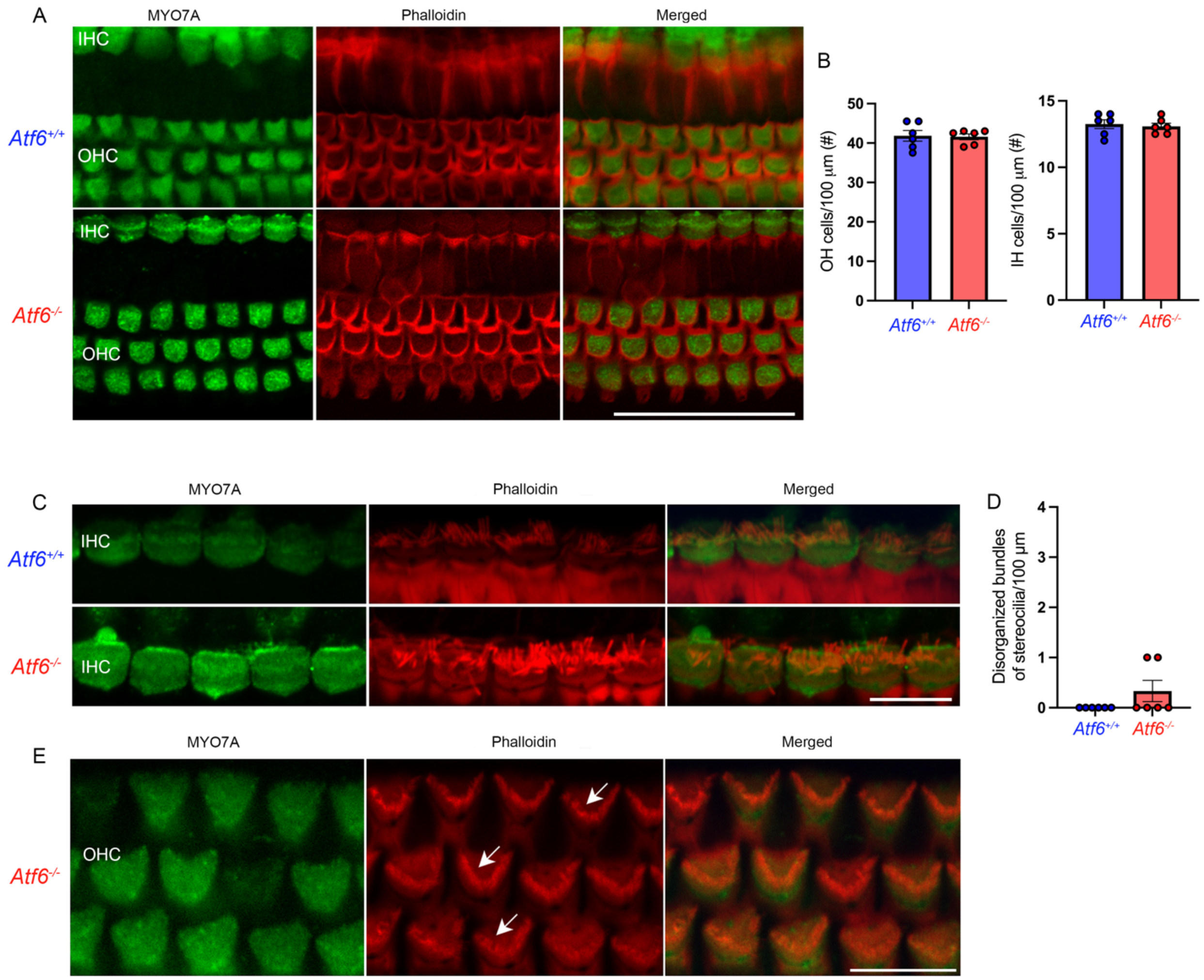

Supplemental Figure 4. Histologic analysis of apical region (approximately 8-12 kHz) of *Atf6*$^{-/-}$ and *Atf6*$^{+/+}$ cochlear hair cells exhibit no loss of hair cells or stereocilia defects. *Atf6*$^{+/+}$ and *Atf6*$^{-/-}$ organ of Corti was stained for myosin 7a (MYO7A) and phalloidin to visualize the hair cells and stereocilia, respectively. (A) Immunofluorescent confocal images of 2-month-old *Atf6*$^{+/+}$ and *Atf6*$^{-/-}$ apical hair cells show no difference in arrangement. (B) Histogram shows no difference in number of apical OHCs and IHCs in 2-month-old *Atf6*$^{+/+}$ (n = 6) and *Atf6*$^{-/-}$ (n=6) cochlea. Counts refer to the number of hair cells encountered within the average of two separate 100 um linear extensions. (Each dot represents the average of two individual measurements; Data represents mean ± SEM, *Welch's t-test,* $**p \leq 0.01$). (C) Histology of cochlear inner hair cell bundles from

Atf6$^{+/+}$ and *Atf6*$^{-/-}$ mice show no differences in the apical region. (D) Quantitative analysis of IHC stereocilia bundle from *Atf6*$^{+/+}$ and *Atf6*$^{-/-}$ mice (Dots represent individual measurements; Data represents mean ± SEM, *Welch's t-test,* $p > 0.05$; n=6/group). (E) Histology of cochlear outer hair cell bundles of *Atf6*$^{-/-}$ mice show intact hair cell bundles in the apical region. OHC, outer hair cell; IHC, inner hair cell. A, Scale bar, 50 um; C, Scale bar, 10 um; E, Scale bar, 20 um.

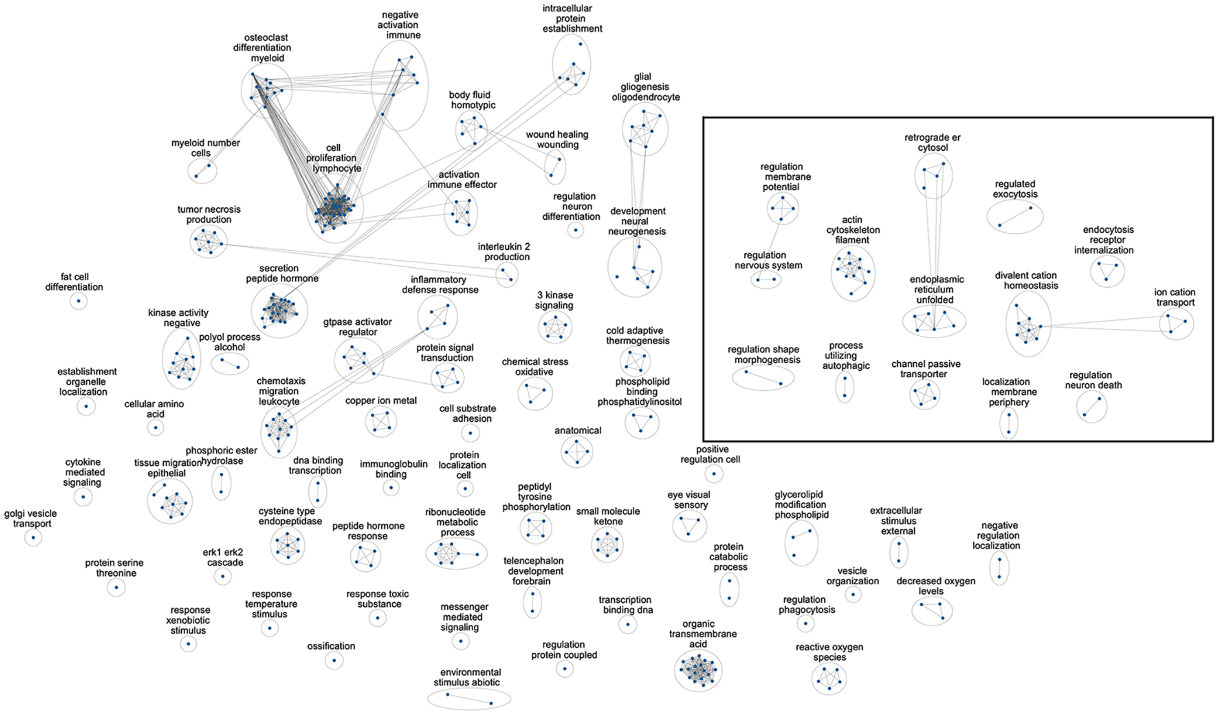

Supplemental Figure 5. Full enrichment map of enriched GO terms from the *Atf6*$^{-/-}$ differentially expressed genes. Visualization of the correlation network is achieved using Cytoscape for GO biological process and GO molecular functions processes associated with the differentially expressed genes (i.e., 1,869 genes). Each dot (blue) is a node that represents a single term from the 2 databases. Individual nodes are clustered into networks (black circles) and labeled by the Cytoscape plug-in, Auto Annotate. Networks related to ER stress, cation transport, actin filaments, channels, neuronal death, and cation homeostasis were presented in black box (Figure 5C).

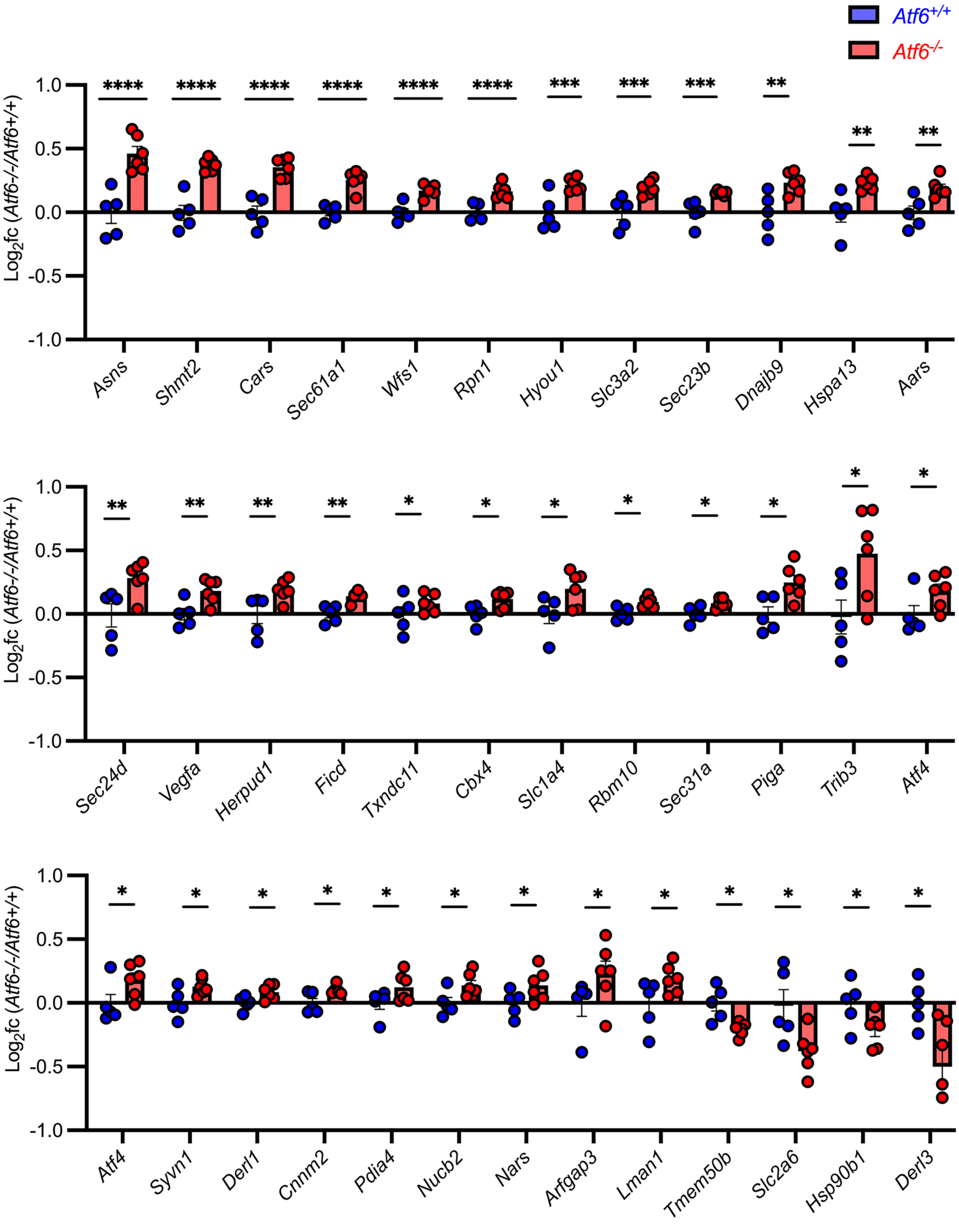

Supplemental Figure 6. Within the UPR-regulated 118 gene panel, 36 genes show statistically significant changes between *Atf6*$^{-/-}$ vs *Atf6*$^{+/+}$ cochleae. Of the 36 genes, 32 are statistically

significantly up-regulated in *Atf6$^{-/-}$* cochlea and 4 are statistically significantly down-regulated in *Atf6$^{-/-}$* cochlea (**** *p ≤ 0.0001,* *** *p ≤ 0.001,* ** *p ≤ 0.01,* * *p ≤ 0.05,* measured from DESeq2 analysis, Supplemental Table 4).

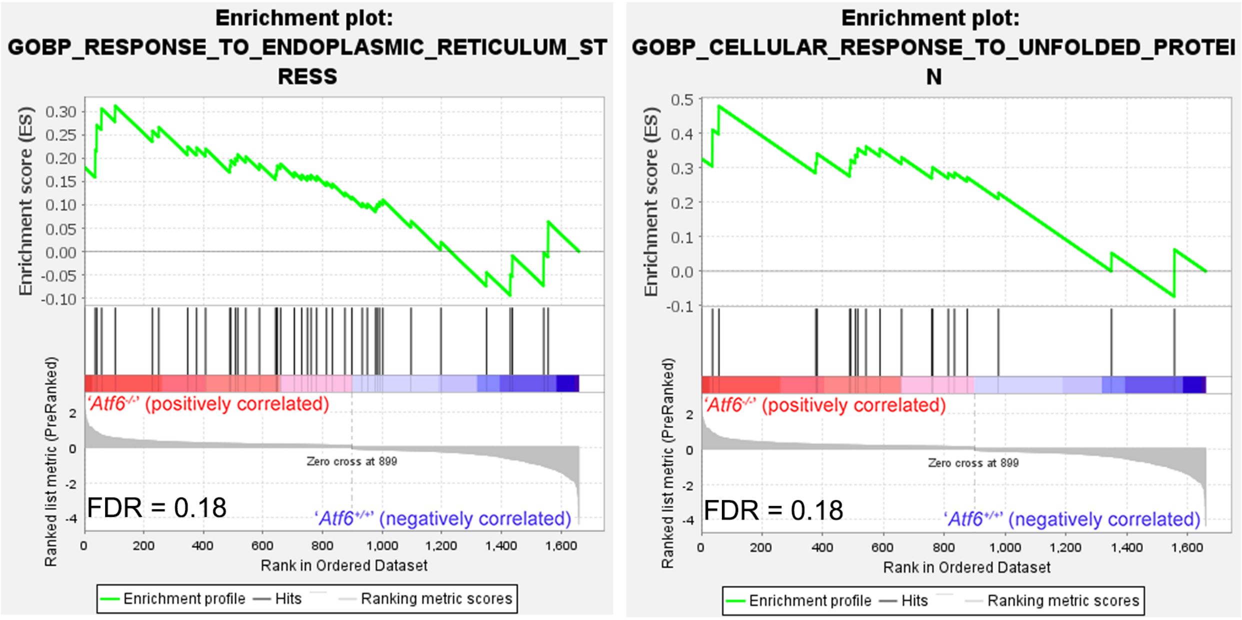

Supplemental Figure 7. Gene set enrichment analysis (GSEA) plots of response to endoplasmic reticulum stress and cellular response to unfolded protein between *Atf6$^{-/-}$* and *Atf6$^{+/+}$* cochlea, with False Discovery Rate (FDR) as indicated. For each plot, the upper part shows the enrichment score and the lower part shows ranked list metric of the gene set. The middle part shows the ranked gene list, with red meaning upregulation (positively correlated), blue for downregulation (negatively correlated), and black vertical line for the genes of the set.

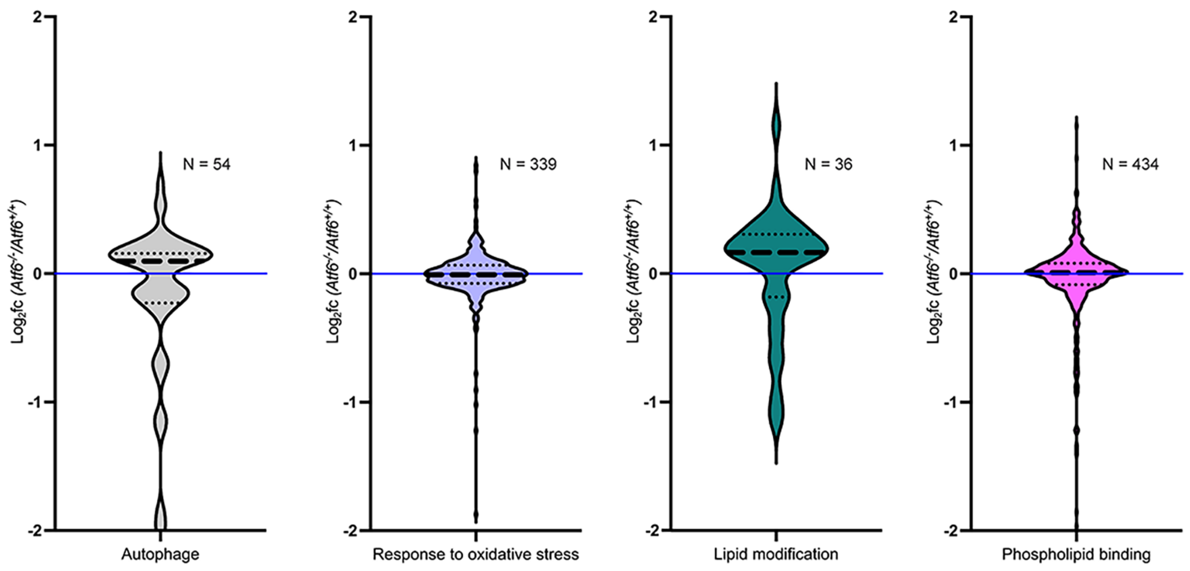

Supplemental Figure 8. No changes in genes related to autophagy, oxidative stress, and lipid synthesis/metabolism in the *Atf6*$^{-/-}$ cochlear transcriptome. Violin plots (gray) show expression levels of 54 autophagy related genes (GO:0006914), violin plots (blue) show expression levels of 339 response to oxidative stress related genes (GO:0006979), violin plots (green) show expression levels of 36 lipid modification related genes (GO:0030258), and violin plots (pink) show expression levels of 434 phospholipid binding related genes (GO:0005543) in *Atf6*$^{-/-}$ cochlea transcriptomes, expressed as log_2fold change relative to *Atf6*$^{+/+}$ cochlea transcriptomes. The complete gene sets are shown in Supplemental Table 7 (*$p > 0.05$, Two-tailed Wilcoxon Signed Rank Test)*. The thick dashed horizontal line represents the median log_2fold change of all genes in the violin plot; and the thin dashed horizontal lines delimit the top and bottom gene quartiles. The horizontal blue line shows no log2fold change.

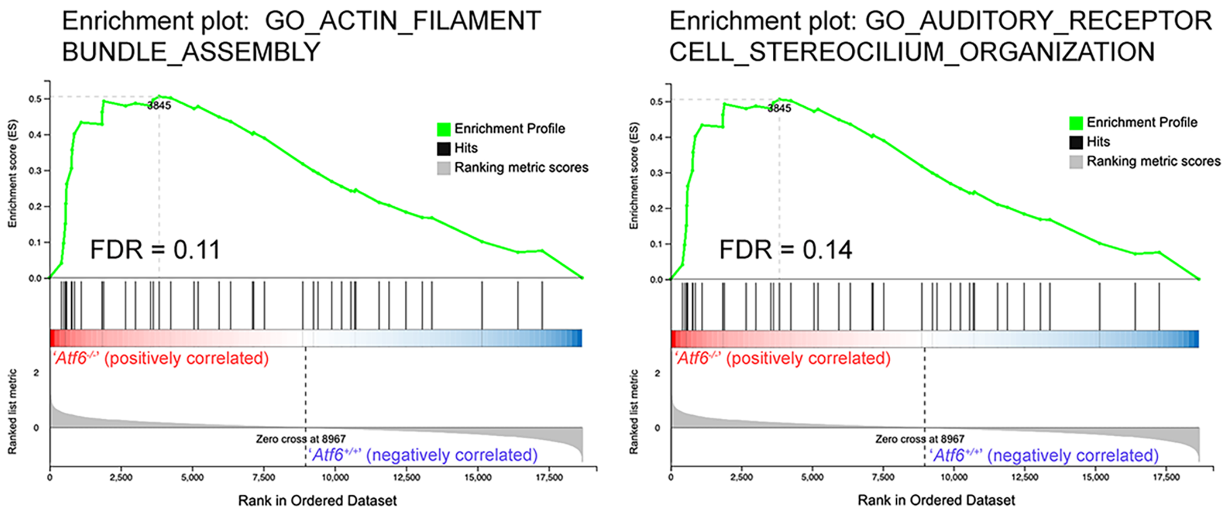

Supplemental Figure 9. Gene set enrichment analysis (GSEA) plots of actin filament organization and hair cell cilium function between *Atf6*$^{-/-}$ and *Atf6*$^{+/+}$ cochlea, with False Discovery Rate (FDR) as indicated. For each plot, the upper part shows the enrichment score and the lower part shows ranked list metric of the gene set. The middle part shows the ranked gene list, with red meaning upregulation (positively correlated), blue for downregulation (negatively correlated), and black vertical line for the genes of the set.

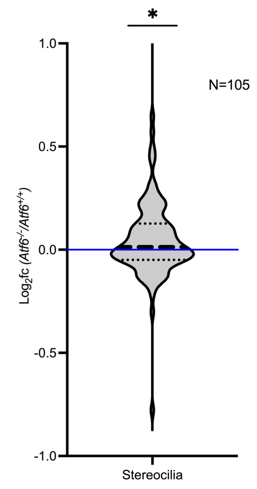

Supplemental Figure 10. Up-regulation of stereocilia genes in *Atf6*$^{-/-}$ cochlea. Violin plots (gray) show expression levels of 105 stereocilia genes in *Atf6*$^{-/-}$ cochlea transcriptomes, expressed as $\log_2$fold change relative to *Atf6*$^{+/+}$ cochlea transcriptomes. The complete gene sets are shown in

Supplemental Table 8 (**p* $>$ *0.05, Two-tailed Wilcoxon Signed Rank Test)*. The thick dashed horizontal line represents the median $\log_2$fold change of all genes in the violin plot; and the thin dashed horizontal lines delimit the top and bottom gene quartiles. The horizontal blue line shows no log2fold change.

Supplemental Table

Supplemental Table 1. List of medical genetic testing hearing loss genes.

Supplemental Table 2. Differential expression (DESeq2) analysis (i.e., based on a cutoff for background value at 0.1 FPKM) from *Atf6*$^{+/+}$ (n =5) and *Atf6*$^{-/-}$ (n=6) cochlea.

Supplemental Table 3. Gene ontology (GO) analysis and Cytoscape visualization of differentially expressed genes identified by RNA-seq.* GO ID: http://geneontology.org

Supplemental Table 4. List of UPR genes from RNA-seq analysis.

Supplemental Table 5. List of ERAD genes from RNA-seq analysis.

Supplemental Table 6. List of intrinsic apoptotic pathway response to ER stress genes from RNA-seq analysis.

Supplemental Table 7. List of autophagy, lipid, oxidative stress, and phospholipid binding genes from RNA-seq analysis.

Supplemental Table 8. List of actin filament and stereocilia-related genes from RNA-seq analysis.

Supplemental Table 9. List of channel-related genes from RNA-seq analysis.
